# Individually redundant effectors are collectively required for bacterial pathogen virulence

**DOI:** 10.1093/ismejo/wraf262

**Published:** 2025-11-26

**Authors:** Lauren M Hemara, Mark T Andersen, Haileigh R Patterson, Marion Wood, Matthew D Templeton, Jay Jayaraman

**Affiliations:** School of Biological Sciences, The University of Auckland, Auckland 1010, New Zealand; The New Zealand Institute for Bioeconomy Science Limited, Mount Albert Research Centre, Auckland 1025, New Zealand; The New Zealand Institute for Bioeconomy Science Limited, Mount Albert Research Centre, Auckland 1025, New Zealand; School of Biological Sciences, The University of Auckland, Auckland 1010, New Zealand; The New Zealand Institute for Bioeconomy Science Limited, Mount Albert Research Centre, Auckland 1025, New Zealand; The New Zealand Institute for Bioeconomy Science Limited, Mount Albert Research Centre, Auckland 1025, New Zealand; School of Biological Sciences, The University of Auckland, Auckland 1010, New Zealand; The New Zealand Institute for Bioeconomy Science Limited, Mount Albert Research Centre, Auckland 1025, New Zealand; Bioprotection Aotearoa, Lincoln University, Lincoln 7647, New Zealand; The New Zealand Institute for Bioeconomy Science Limited, Mount Albert Research Centre, Auckland 1025, New Zealand

**Keywords:** pseudomonas syringae pv. actinidiae, plant-pathogen interactions, bacterial virulence

## Abstract

Host specificity of a plant pathogen is defined by its effector complement. However, it remains unclear whether the full complement is required for pathogenicity. *Pseudomonas syringae* pv. *actinidiae (*Psa) is an emerging model pathogen of kiwifruit with over 30 functional effectors, providing a unique opportunity to understand how host-mediated selection shapes pathogen evolution. The majority of Psa’s effectors previously appeared nonessential in single knockout contexts. Why, then, does Psa maintain such a large repertoire? We sought to examine effector requirements, redundancies, and repertoire refinement across host genotypes through a mutated effector-leveraging evolution experiment (MELEE), serially passaging competitive populations of effector knockout strains. Competition suggests that all effectors are collectively required for successful virulence, demonstrated by the dominance of wild-type. Host-specific effector requirements identified may further explain the maintenance of this large effector repertoire, providing important insights into the dynamics of effector redundancy following incursions.

## Introduction

Gram-negative bacteria secrete effectors into host cells through the type III secretion system (T3SS), where they interact with host targets to aid pathogen entry, facilitate nutrient extraction, and subvert host immunity [1–5]. However, effectors can also be recognised by host resistance proteins, activating effector-triggered immunity (ETI) [6–9]. Pathogens are, therefore, under selective pressure to refine their effector complement, balancing the retention of effectors required for virulence with the loss of recognised effectors that elicit immunity. However, it is currently unclear whether pathogens require their full effector complement for virulence or if only a subset is necessary.

Virulence, the ability to infect and express symptoms in susceptible hosts, is considered an emergent property which cannot be explained in totality by its individual components [10]. In the context of effector repertoires, the collective repertoire function is considered greater than the additive effects of each individual effector [11]. To date, effector research has focused on the pathogenicity of single- and poly-effector mutants [3, 12–23]. However, even with complete knowledge of individual effector functions or virulence contributions, we know little of their interplay and connectivity [11, 24]. To better understand virulence, a shift away from individual effector dissection and towards perturbation of effector repertoires in community contexts is required.

Recent research has demonstrated the ability of effectors to act as public goods, compensating *in trans* for other strains that lack a given effector. The collective virulence of disaggregated effector repertoires has been demonstrated on *Arabidopsis*, across a co-isogenic *P. syringae* pv. *tomato (*Pto) population (termed a `metaclone') [25]. More generally, the ability for individually nonpathogenic or avirulent strains or subpopulations to freeload off of pathogenic strains has been observed both in *P. syringae* and other pathosystems [25–30]. Conversely, two recent studies in mammalian pathosystems have developed single-cell profiling methodologies to measure the impact of individual effector loss in community settings [31, 32]. Pooled CRISPR-knockout screening, combined with single-cell profiling, established critical roles for *Toxoplasma gondii* effectors during infection that were not previously identified in growth-based knockout screens *in vivo* [31, 33–36]*.* Similarly, multiplexed bacterial barcodes tracked *Salmonella typhimurium* effector mutants during infection, identifying mutations in effectors from *Salmonella* Pathogenicity Island 2 (SPI-2) which led to mutant-specific expression patterns [32]. These SPI-2 effectors form a complex network, with *S. typhimurium* able to tolerate single effector deletions without affecting virulence, underscoring the redundancy in this secreted repertoire (Ori *et al.*, 2023). A key challenge remains in understanding effector requirements, redundancy, and interplay at the repertoire level, given this potential for both refinement and co-operative virulence.

*P. syringae* pv. *actinidiae* biovar 3 (Psa3) is an emerging model pathogen of kiwifruit with a large effector repertoire, presenting a unique opportunity to study the role of host-mediated selection following disease outbreaks. Psa3 effector knockout pathogenicity assays indicate that only a few effectors are required for virulence on the susceptible kiwifruit cultivar *Actinidia chinensis* var. *chinensis* `Hort16A'*—*HopR1b, AvrE1d, HopAZ1a, and HopS2b [15, 16]. Two further redundant effector groups are involved in immune suppression [16]. Furthermore, several effectors are recognised by resistant kiwifruit hosts [14, 37]. Despite this recognition, genome biosurveillance of orchard-based populations suggests that repertoire refinement is rare, with only a few documented instances of effector loss emerging [14, 37, 38]. Why then, does Psa3 retain so many effectors in its repertoire? Ostensibly, if only a few effectors make major contributions to virulence, and if hosts recognise other nonessential effectors, there is little benefit to maintaining a large effector repertoire. It could be that genetic diversity in the host population selects for the retention of a large effector repertoire, with each host exerting a distinct selection pressure. It may be that effector loss is simply rare and thus most effectors are retained. Alternatively, effectors presumed to be `nonessential' may be retained due to virulence contributions that cannot be detected through classical pathogenicity assays. Given the lack of repertoire refinement in the field, this research sought to understand the potential consequences of repertoire refinement in a controlled experimental setting. Furthermore, rather than relying upon mutations emerging in a wild-type background, we have preloaded this system with mutations of interest to comprehensively observe how selection acts upon effector loss across Psa3’s repertoire.

## Materials and methods

### Microbiological methods

All Psa strains were streaked from glycerol stocks onto LB agar supplemented with 12.5 μg/ml nitrofurantoin (Sigma Aldrich, New Zealand) and 40 μg/ml cephalexin (Sigma Aldrich) for Psa selection. To select for Psa strains carrying pBBR1MCS-5B vectors for effector complementation, LB agar was supplemented with 50 μg/ml gentamicin (Sigma Aldrich). Plates were sealed with parafilm and grown for 48 h at 22°C. LB cultures were grown overnight on a digital orbital shaker at 100 rpm and 22°C.

Psa3 V-13 knockout strains and Psa3 V-13 ∆*33E* plasmid-complemented strains [39] are described in Supplementary Table 1. These strains were transformed as described in Jayaraman *et al.* [15].

#### *In vitro* serial passaging

Psa3 V-13 knockout strains (Supplementary Table 1) were grown overnight in 5 ml LB and pooled together in equal proportion, with the final pooled population diluted to a total OD_600_ of 0.005 in 500 ml 10 mM MgSO_4_. 10 μL of this pool was used to inoculate either 10 ml LB or 10 ml minimal media [40] in 50 ml falcon tubes, with three replicates. The LB was shaken on an orbital shaker at 100 rpm for 48 h. 10 μL of culture was then sampled and used to inoculate a fresh aliquot of 10 ml LB. For each replicate, *in vitro* passaging was conducted for three passages across 6 days total.

### *In planta* pathogenicity and competition assays

#### Tissue culture plantlets

*Actinidia* spp. tissue culture plantlets were supplied by MultiFlora Laboratories (Auckland, New Zealand) and *Malus* tissue culture plantlets were supplied by Plant & Food Research. *Actinidia* plantlets were grown in 400 ml lidded plastic pottles on half-strength Murashige and Skoog (MS) agar, with three plantlets per pottle for *A. chinensis* var. *chinensis* `Hort16A' and *A. chinensis* var. *deliciosa* `Hayward', and five plantlets per pottle for *A. arguta* AA07_03. *M. domestica* `Royal Gala' tissue culture plantlets were grown on half-strength MS agar supplemented with 6-benzylaminopurine and indole-3-butyric acid, with two plantlets per pottle.

#### Flood inoculation

Tissue culture plantlets were flood inoculated with Psa at an OD_600_ of 0.005 using the pathogenicity assay established in McAtee *et al.* [41]. Briefly, *Actinidia* tissue culture plantlets were flooded with 500 ml Psa inoculum (10^6^ CFUs/ml) suspended in 10 mM sterile MgSO_4_ for 3 min. Plantlets were grown in a climate control room at 20°C with a 16 h/8 h light/dark cycle. Bacterial growth was quantified at 12 days post-inoculation (dpi) by quantitative polymerase chain reaction (qPCR) or plate count quantification [14].

#### Vacuum infiltration

Vacuum infiltration was performed using a Rocker 400 oil-free vacuum pump and glass bell. For each treatment, the bacterial inoculum was normalised to an OD_600_ of 0.005 in 500 ml 10 mM MgSO_4_, pottles were flooded, and the inoculum was then vacuum infiltrated into *A. chinensis* var. *chinensis* `Hort16A' tissue culture plantlets. Tissue culture pottles were held at ~80 kPa (600 mmHg) for two 1 min bursts, with the bell depressurised and reset between each burst.

#### *In planta* competition and serial passaging assays

Psa3 V-13 knockout strains (Supplementary Table 1) were grown overnight in 5 ml LB and pooled together in equal proportion based on OD, with the final pooled population diluted to a total OD_600_ of 0.005 in 500 ml 10 mM MgSO_4_. This population was used to flood inoculate three replicate tissue culture pottle `lineages' per host genotype.

To quantify the relative bacterial growth of each strain *in planta*, leaf discs were harvested 12 days post-inoculation. A 0.8 cm diameter cork borer was used to punch 16 leaf discs per pottle. Leaf discs were briefly washed in 40 ml of sterile MilliQ H_2_O. Four technical replicates of four leaf discs each were sampled evenly from the plantlets in each pottle, with each technical replicate ground in 350 μL sterile 10 mM MgSO_4_ with three 3.5 mm stainless steel beads in a Storm24 Bullet Blender (Next Advance, NY, USA). Samples were ground twice at maximum speed for 1 min. A further 350 μL sterile 10 mM MgSO_4_ was added, and samples were ground at maximum speed for 1 min.

To recover the bacterial population, the resulting leaf homogenate was used to inoculate 50 ml LB supplemented with 12.5 μg/ml nitrofurantoin in a 500 ml conical flask. Leaf homogenate inocula of 200 μL, 300 μL, or 600 μL was used for *A. chinensis* var. *chinensis* `Hort16A', *A. chinensis* var. *deliciosa* `Hayward', or *A. arguta* AA07_03 and *M. domestica* `Royal Gala', respectively. This amount was increased for tolerant and resistant accessions, as less bacterial inoculum was recovered. Leaf sampling was adapted for *M. domestica* `Royal Gala', as the leaf material was smaller than the cork borer used to punch leaf discs. For `Royal Gala' tissue culture plantlets, 20 mg of leaf material was sampled evenly across plantlets from each tissue culture pottle. Flasks were shaken on a digital orbital shaker at 100 rpm for 48 h.

Aliquots (1 ml) of bacterial culture were sampled after shaking for DNA extraction, long term glycerol stock storage, and serial passaging (where applicable). At each passage, sterile tissue culture plants were flood inoculated with the newly recovered bacterial population at an OD_600_ of 0.005 and incubated for another 12 days. DNA was extracted using a Qiagen DNeasy Blood & Tissue Kit (Qiagen, Hilden, Germany), following the Gram-negative bacteria protocol. Quantitative polymerase chain reaction (qPCR) was conducted using strain-specific primers.

#### Metaclone assembly

Psa3 V-13 metaclones were assembled according to a previously established method [25], with the effector-carrying Psa3 ∆*33E* + pBBR1MCS-5 strains in Supplementary Table 1. A metaclone is a coisogenic population, where each strain in the population is identical expect for a single loci—the presence of a single effector in an otherwise effectorless background [25]. These strains were combined in equal proportion to a total OD_600_ of 0.005 and used to inoculate *A. chinensis* var. *chinensis* `Hort16A' tissue culture pottles through vacuum infiltration. Bacterial growth was measured at 12 dpi by plate count.

### DNA extraction

For DNA extraction from leaf tissue, ground tissue homogenate was stored overnight at −20°C prior to extraction with the PDQeX platform [42] (MicroGEM, Dunedin, New Zealand). For DNA extraction from LB culture, the Qiagen DNAeasy Blood & Tissue kit was used for DNA extractions, following the protocol for Gram-negative bacteria (Qiagen, Hilden, Germany). DNA was diluted 10-fold before being used as templates for quantitative PCR.

### Effector knockout tracking primer design

Primers were designed to amplify a short product exclusively from the effector knockout locus of each effector knockout strain, across the *XbaI* site introduced during pK18mobsacB cloning. Oligonucleotides were synthesised by Macrogen (South Korea). Primers were resuspended in MilliQ water to a concentration of 0.1 mM and stored at −20°C. Working primer solutions were diluted to a concentration of 5 μM. qPCR primers are listed in Supplementary Table 2. Primer exclusivity was assessed using DNA extractions from wild-type Psa3 and relevant effector knockouts. Primer efficiency was assessed using serial dilutions of gDNA extracted from the original competitive population of Psa3 effector knockout strains pooled in equal proportion.

### Construction of partial and full virulence-required knock-in strains

For the partial virulence-required knock-in (KI) strain, KI constructs were synthesized (GenScript, Singapore) for *hopR1b*, a single module for PTI suppression (PTI-E: *hopAW1a* and *hopD2a*), a single module for RIN-targeting effectors (RIN4-E: *hopZ5a* and *hopH1a*), and a joint single module for individually required effectors (Individual-E: *hopAZ1a* and *hopS2b*) identified in previous research [16]. All effectors were synthesized under control of their own independent native promoter. These modules were cloned into the original pK18mobsacB backgrounds used to knockout these effectors [14, 15], except for the PTI-E module which was cloned into pK18mobsacB:Δ*avrRpm1a*. The KI construct for *avrE1d* was generated previously [15]. The Psa3 V-13 Δ*33E* strain was used to knock-in these five aforementioned effector modules (AvrE1d, HopR1b, PTI-E, RIN4-E, and Individual-E).

For the full virulence-required KI strain, a Psa3 V-13 Δ*28E* strain, retaining some virulence-required effectors identified from serial passaging (*hopAM1a* and *hopI1c*), was used. In addition to the five effector loci knocked into the partial virulence-required KI strain, a construct for *hopBP1a* was also synthesized (GenScript) and cloned into the original knockout vector to generate the full virulence-required KI strain [14]. Additionally, *hopAS1b* was complemented on a pBBR1MCS-5 plasmid cloned previously [39].

### Pathogenicity testing knock-in strains

Pathogenicity of knock-in strains was tested using flood inoculation and relative quantification as previously described [14].

### Quantitative PCR

Real-time qPCR was performed using an Illumina Eco Real-Time PCR platform, following the protocol developed by Andersen *et al.* [43]. Bacterial growth was assessed by relative quantification. The cycle threshold (Ct) value for each knockout primer pair (Supplementary Table 2) was normalised using the ∆∆Ct method—first to the Psa ITS Ct value from the same generation (∆Ct_ITS_ - ∆Ct_KO_), and then to the ∆Ct values for the original inoculum (∆Ct_inoculum_ - ∆Ct_generation_). Relative quantification values were visualised as 2^-∆∆Ct^.

### De novo variant identification in serially passaged Psa3 populations

Sequencing libraries were constructed and sequenced on a NovaSeqX System (Illumina; paired-end 150 bp reads) by the Australian Genome Research Facility (AGRF, Melbourne, Australia). Breseq [44] (version 0.38.1) was used in polymorphism mode to determine the frequency of variants over generations one, three, and nine for all lineages of the Psa3 competitive effector knockout pool. The original inoculum population was also analysed as a control. Sequence data are available in the Sequence Read Archive (https://www.ncbi.nlm.nih.gov/sra) under BioProject PRJNA1330500.

### Protein structural prediction

Protein structures were predicted from amino acid sequences using ColabFold v1.5.5 [45] with default settings (msa_mode: mmseqs2_uniref_env, pair_mode: unpaired_paired, model_type: auto, num_recycles: 3, recycle_early_stop_tolerance: auto, relax_max_iterations: 200, pairing strategy: greedy, max_msa: auto, num_seeds: 1). Protein structures were visualised in ChimeraX [46].

### Data visualisation and statistical analysis

Statistical analysis was conducted in R [47], and figures were produced using the packages ggplot2 ([48]; version 3.5.2) and ggpubr ([49]; version 0.3.0). Plots were exported from R as PDF files and prepared for publication in Adobe Illustrator (Adobe Inc.). Post-hoc statistical tests were conducted using the ggpubr ([49]; version 0.3.0), agricolae ([50]; version 1.3), and PMCMRplus ([51]; version 1.9.12) packages. A Shapiro test was used to assess normality. If the Shapiro-Wilks test indicated that a given population was normally distributed, the stats_compare_means() function from the ggpubr package was used to calculate omnibus one-way analysis of variance (ANOVA) statistics to identify significant differences across all treatment groups [49]. If the Shapiro-Wilks test indicated that a given population was significantly different from a normal distribution, a nonparametric Kruskal–Wallis test was conducted. Datasets with significant ANOVA or Kruskal–Wallis *P*-values proceeded to post-hoc statistical tests. For normally-distributed populations, Welch’s t-test was used to conduct pair-wise, parametric t-tests between an indicated group and a designated reference [49]. For non-normal distributions, a Wilcoxon test was used to conduct pair-wise, nonparametric tests between an indicated strain and a designated reference strain [49], and the kwAllPairsNemenyiTest() function from the PMCMRplus package was used to perform Nemenyi’s nonparametric all-pairs comparison test with a Bonferroni *P*-value adjustment ([51]; version 1.9.12).

Data was normalised with the scale() function and a principal components analysis (PCA) was performed with the princomp() function. PCA plots were created with the fviz_pca_biplot() and fviz_pca_ind() functions from the factoextra package (version 1.0.7; [52]).

Graphical schematics were made in BioRender (https://www.biorender.com/).

## Results

### Individually redundant effectors are collectively required for virulence

**Figure 1:**
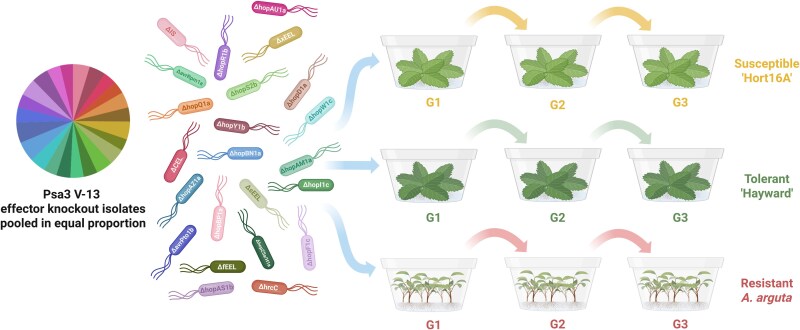
MELEE (mutated effector-leveraging evolution experiment) methodology. Psa-susceptible *A. chinensis* var. *chinensis* `Hort16A', Psa-tolerant *A. chinensis* var. *deliciosa* `Hayward' and Psa-resistant *A. arguta* AA07_03 were infected with a competitive population of Psa3 effector knockout strains. These populations were then passaged across plant generations at 12 day intervals post-inoculation. Unique primers were designed for each effector knockout over the *Xba*I site (introduced through knockout cloning) to track the corresponding effector knockout strain. The pie chart shows the ∆Ct value for each effector knockout strain normalised to Psa ITS in the initial inoculum pool.

A mutated effector-leveraging evolution experiment (MELEE) was designed, competing effector knockout strains against one another over serial passages to assess the impact of effector loss on virulence and *in planta* growth in a population setting. We hypothesised that the selective pressure exerted by competition, combined with the narrow bottlenecks produced by passaging, would allow more sensitive detection of subtle virulence contributions. Individual Psa3 effector knockout strains, representing all of Psa3’s functional effectors, were pooled in equal proportion. This population also contained two control strains. “Wild-type” ∆*IS* (WT∆*IS*) carries Psa3’s full effector repertoire, with a redundant insertion sequence (IS) knocked out which does not contribute to virulence (Fig. S1), whereas avirulent Psa3 ∆*hrcC* cannot produce a functional T3SS. This pool mimics the natural emergence of mutations in the field, as each mutant is infrequent in the population (1/23) unless acted upon by selection. These pools were passaged across susceptible *A. chinensis* var. *chinensis* `Hort16A', tolerant *A. chinensis* var. *deliciosa* `Hayward' (able to resist the effects of Psa infection without limiting pathogen growth), and resistant *A. arguta* AA07_03 (able to limit pathogen growth *in planta*), representing a spectrum of potential Psa3 infection outcomes (Fig. 1; [53–56]).

**Figure 2:**
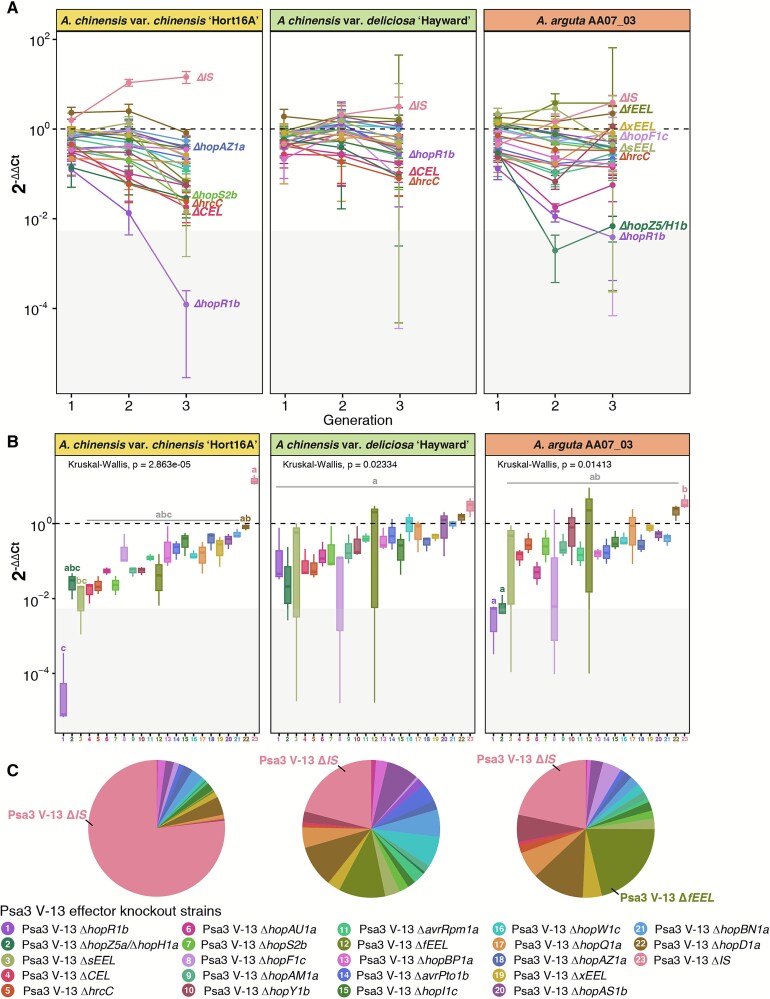
Three generations of serial passaging reveals that the majority of effector knockouts are selected against in a host-specific manner. Three generations of a competitive Psa3 effector knockout population were serially passaged across *A. chinensis* var. *chinensis* `Hort16A', *A. chinensis* var. *deliciosa* `Hayward', and *A. arguta* AA07_03 tissue culture plantlets. The dashed line represents a 2^-∆∆Ct^ value of 1. Strains above this line have increased relative to the starting population, whereas strains below this line have decreased. The grey box highlights the range of 2^-∆∆Ct^ values that correspond to raw Ct values of 40, indicating that the effector knockout strain could not be detected by qPCR. (A) the line graph shows the relative abundance of effector knockout strains over time, normalised to Psa ITS for each generation and the starting population. The round point represents the mean and the error bars represent the standard error across three replicate lineages. Key knockout strains are annotated. (B) The boxplots show the relative abundance of effector knockout strains at generation 3, normalised to Psa ITS for each generation and the starting population. Effector knockout strains with different letters are significantly different at α ≤ 0.05, as determined by Nemenyi’s nonparametric all-pairs comparison test, performed individually for each host. (C) The pie charts show the mean 2^-∆∆Ct^ value at generation 3 for each effector knockout strain for *A. chinensis* var. *chinensis* `Hort16A', *A. chinensis* var. *deliciosa* `Hayward', and *A. arguta* AA07_03.

WT∆*IS* emerged as the dominant isolate across independent replicates following three generations of passaging, albeit without statistical significance due to high variability across populations (Fig. 2). This dominance was particularly visible on susceptible `Hort16A', where all effector knockout strains decreased in the population over time (Fig. 2), despite most lacking a demonstrable virulence contribution in individual pathogenicity assays (Fig. S2). Moreover, some effector knockout strains dropped out of the population to a greater extent than others (Fig. 2). ∆*hopR1b* dropped out the furthest, further than the `avirulent' control ∆*hrcC* and beyond the qPCR detection limit. Curiously, ∆*CEL*, lacking AvrE1d, did not drop out to the same extent, despite a similar individual virulence contribution [15]. Conversely, some knockout strains with known virulence contributions performed better than expected. HopAZ1a makes a minor virulence contribution on `Hort16A' [16]. However, the corresponding knockout strain did not decrease in the population beyond a 2^-∆∆Ct^ of 10^−1^, on par with knockouts of effectors that are not known to make independent virulence contributions (Fig. 2, Fig. S2). HopS2b also makes a similar minor contribution [16], yet the reciprocal knockout dropped out further (Fig. 2, Fig. S2). The differences that emerged between these major and minor virulence effector knockouts could also be replicated in a smaller competitive pool (Fig. S3).

Although the dominance of WT∆*IS* was most pronounced on `Hort16A', it was also the fittest strain on tolerant `Hayward' and resistant *A. arguta* (Fig. 2). Host-specific fates were also observed for some effector knockouts (Fig. 2). On `Hayward', Δ*hopAZ1a*, Δ*fEEL*, Δ*hopBN1a*, Δ*sEEL*, and WTΔ*IS* all increased in the population over time, whereas on *A. arguta* only Δ*fEEL* and WTΔ*IS* increased (Fig. 2). Individually, avirulence effector knockouts – such as the exchangeable effector locus (EEL) effector *hopAW1a* – can partially escape *A. arguta*’s immunity [14]. We have previously generated a number of knockouts in the EEL, resulting in `short' (sEEL), `full' (fEEL), and `extended' (xEEL) deletions of different effector combinations, all of which knockout recognised *hopAW1a* ([14]; Fig. S2). However, the loss of recognised effectors appeared to be detrimental in a mixed, ETI-eliciting population on a resistant host. These strains may be trapped by a virulence trade-off, losing a contribution to virulence without escaping the burden of recognition, such that WT∆*IS* was the fittest genotype despite eliciting ETI. This was exemplified by the wide distribution of Δ*fEEL* across independent lineages sampled from *A. arguta (*Fig. 2). Although Δ*fEEL* was one of the few strains to increase in the population over time, in one lineage this knockout dropped the furthest of any strain (Fig. 2B). Given that this ′full′ EEL deletion removed numerous effectors [14], these strains appeared to precariously balance virulence requirements with recognition evasion in competition, making them vulnerable to sudden loss of virulence; a phenomenon also seen for the ′small′ EEL knockout Δ*sEEL* and Δ*hopF1c*. The more extensive EEL knockout Δ*xEEL* appeared less vulnerable to these sudden drops (Fig. 2). This EEL deletion strain lacks *hopD1a*, another avirulence effector recognised by all three hosts [14, 16]. Given that Δ*hopD1a* performs relatively well across hosts (Fig. 2B), the benefit of *hopD1a* loss may balance out the cost of losing other EEL effectors. Curiously, the trajectories for these ′precarious′ knockout strains (Δ*hopF1c*, Δ*sEEL* and Δ*fEEL*) across tolerant ′Hayward′ and resistant *A. arguta* mirrored each other, suggesting a shared virulence role or burden of recognition across both hosts [14].

### Changes in population structure during *in planta* passaging are driven by host-specific selection

**Figure 3:**
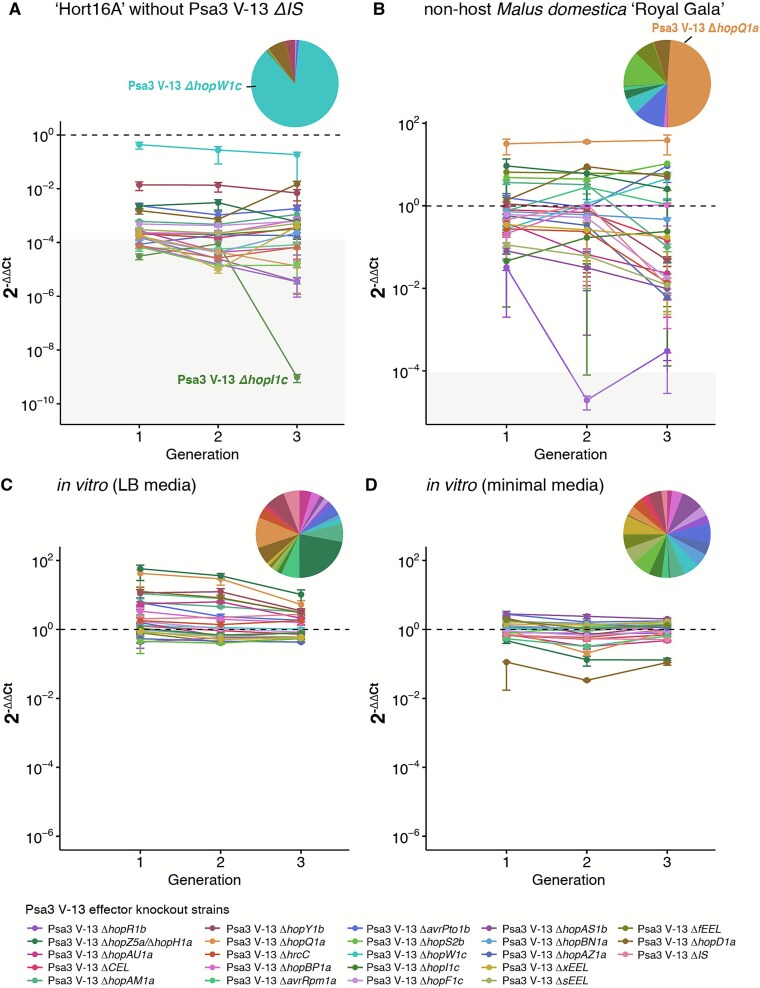
Changes in population structure during *in planta* passaging are driven by host-specific selection, rather than *in vitro* nutritional or growth processes. Three generations of a competitive Psa3 effector knockout population were serially passaged (A) without the wild-type ∆*IS* strain on *A. chinensis* var. *chinensis* `Hort16A', (B) on the nonhost *Malus domestica* `Royal Gala', (C) *in vitro* in rich LB media, and (D) *in vitro* in *hrp*-inducing minimal media. The dashed line represents a 2^-∆∆Ct^ value of 1. Strains above this line have increased relative to the starting population, whereas strains below this line have decreased. The grey box highlights the range of 2^-∆∆Ct^ values that correspond to raw Ct values of 40, indicating that the effector knockout strain could not be detected by qPCR. The line graph shows the relative abundance of effector knockout strains over time, normalised to Psa ITS for each generation, and to the starting population. The round point represents the mean and error bars represent standard error across three replicate lineages. The pie charts show the mean 2^-∆∆Ct^ value at generation 3 for each effector knockout strain.

Psa3 WTΔ*IS* appeared to be the fittest strain across kiwifruit hosts (Fig. 2). In the absence of wild-type, are other strains able to dominate the population? To test this, MELEE without WTΔ*IS* was carried out on susceptible ′Hort16A′. Over three passaging generations, there was no domination of individual knockout strains in the absence of the full effector repertoire in a single strain; in fact, no effector knockout strain increased in the population (Fig. 3A). The closest contender was *ΔhopW1c*, while all other knockouts decreased to at least 10^−2^ (Fig. 3A). Understanding the degree to which the host immune response influences population dynamics was also of interest, particularly when considering the phenomenon of ′nonhost′ immunity. Although Psa-resistant *A. arguta* AA07_03 may be considered a nonhost due to its recognition of Psa3 effectors, we next sought to explore the competitive fitness of effector knockout strains on a ′true′ nonhost that Psa does not share an evolutionary history with. The apple (*M. domestica*) cultivar ′Royal Gala′ was selected as an evolutionarily-unrelated nonhost species to identify whether the effector requirements identified on *Actinidia* spp. were genuinely host-specific. MELEE on this nonhost revealed that WT∆*IS* fared poorly on ′Royal Gala′, confirming that Psa3’s effector repertoire is finely tuned for infecting its kiwifruit host and is not compatible with apple (Fig. 3B). Indeed, in stark contrast to passaging on *Actinidia*, WT∆*IS* no longer fared better than the majority of effector knockouts *(*Fig. 3B, Fig. S4). In this context, Δ*hopQ1a* emerged as the dominant strain (Fig. 3B, Fig. S4). Some effector knockouts appeared to be `universally' selected against across *Actinidia* and *Malus—*Δ*hopR1b,* Δ*hrcC,* Δ*hopAZ1a,* Δ*sEEL,* Δ*hopAM1a,* Δ*hopAU1a* and Δ*CEL (*Fig. 2 and 3B, Fig. S4). As with *Actinidia,* Δ*hopR1b* drops out the furthest, whereas *CEL* loss is not selected against to the same extent (Fig. 2 and 3B, Fig. S4).

Finally, to ensure that host genotype was responsible for these competition outcomes, the full competitive pool of Psa3 effector knockout strains was passaged in two *in vitro* environments—rich LB medium (Fig. 3C) or *hrp*-inducing minimal medium (Fig. 3D), which mimicks the leaf apoplastic environment to induce effector secretion [57–59]. Different trajectories were observed between *in vitro* and *in planta* passaging (Fig. 2, Fig. 3C and D). *In vitro*, most strains increased in the population (relative to inoculum) with no strains showing a significant lack of fitness, as none were significantly selected against over successive passages (Fig. 3C and D). Furthermore, the few effector knockout strains which decreased in the population were distinct from the knockouts selected against *in planta*. This suggested that the population changes observed *in planta* were primarily influenced by host selection and *in planta* fitness of these strains, rather than by nutritional deficiencies or growth artefacts.

The outcome of *in planta* competition also appears to depend on the inoculation method. Several Psa effectors, including HopR1b, have been implicated in host entry. Specifically, a Psa3 *hopR1* mutant fails to reopen stomata during plant infection, suggesting that HopR1b facilitates stomatal entry alongside performing other virulence functions like PTI suppression [60]. Could potential functional specialisation explain the differences emerging between Psa’s large pore-forming effectors—particularly AvrE1d and HopR1b [16]—with some having specific roles in host entry at stomatal cells and others apoplastic wetting? In order to evaluate this, vacuum infiltration was used to infect *A. chinensis* var. *chinensis* `Hort16A' plantlets with a competitive effector knockout population, at the standard OD of 0.005. In contrast to flood inoculation, vacuum infiltration of this knockout pool into susceptible `Hort16A' resulted in all knockout strain increasing in the population 12 days-post infection, relative to the starting inoculum—with the sole exception of Psa3 V-13 ∆*hopZ5a*/∆*hopH1a* (Fig. S5).

### Extended passaging reveals host-specific effector requirements

**Figure 4:**
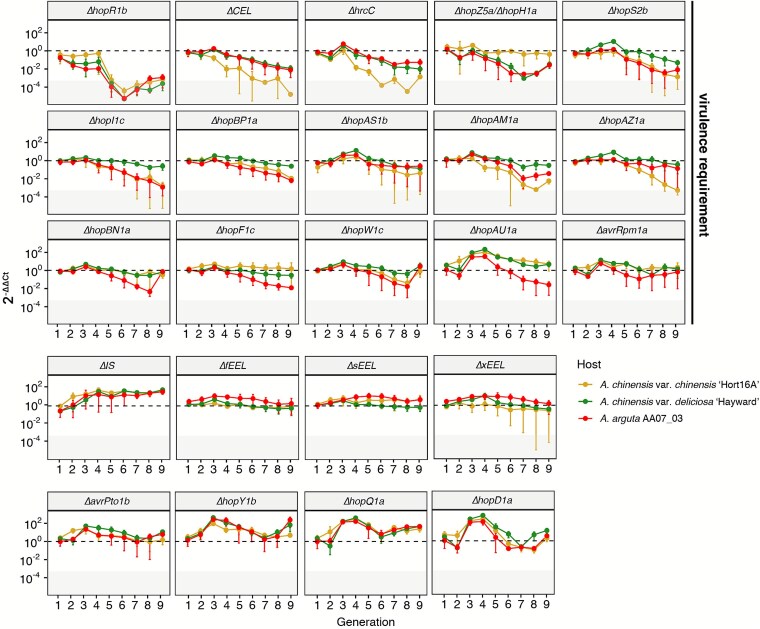
Extended passaging reveals that the majority of effectors have previously unidentified, host-specific virulence requirements. Nine generations of a competitive Psa3 effector knockout population were serially passaged across *A. chinensis* var. *chinensis* `Hort16A', *A. chinensis* var. *deliciosa* `Hayward', and *A. arguta* AA07_03 tissue culture plantlets, faceted by Psa3 effector knockout strain. The dashed line represents a 2^-∆∆Ct^ value of 1. Strains above this line have increased relative to the starting population, whereas strains below this line have decreased. The grey box highlights the range of 2^-∆∆Ct^ values that correspond to raw Ct values of 40, indicating that the effector knockout strain could not be detected by qPCR. The line graph shows the relative abundance of effector knockout strains over time, normalised to Psa ITS for each generation, and to the starting population. The round point represents the mean and error bars represent standard error across three replicate lineages. Effectors with a virulence role, identified by individual pathogenicity or competition assay, are indicated by annotation.

In order to test MELEE reproducibility and better tease apart effector requirements, kiwifruit host passaging was repeated over an extended time-series, for a total of nine generations. Separating the trajectory of each effector knockout strain over time revealed clear profiles (Fig. 4, Fig. S7). Despite a slower start in this extended experiment, the trajectories of key knockout strains (e.g. WTΔ*IS*, Δ*CEL*, Δ*hrcC* and Δ*hopR1b*) were consistent across both kiwifruit passaging experiments (Fig. S6). For the nine-generation experiment, knockout strains of all effectors with known virulence requirements in `Hort16A' decreased over time, including Δ*hopR1b*, Δ*CEL*, Δ*hopS2b*, Δ*hopAZ1a*, Δ*hrcC*, and Δ*xEEL [**15**,*  *16**]* (Fig. 4, Figs S7 and S8). Other effector knockout strains, including Δ*hopZ5a/*Δ*hopH1a,* Δ*hopI1c,* Δ*hopBP1a,* Δ*hopAS1b,* Δ*hopAM1a*, Δ*hopBN1a,* Δ*hopF1c,* Δ*hopAU1a,* Δ*hopW1c,* and *avrRpm1a*, also appeared to be selected against, suggesting previously unidentified virulence contributions (Fig. 4, Figs S7 and S8). In this extended time-series, Δ*hopAZ1a* was also selected against, in line with expectations from `Hort16A' pathogenicity tests [16] (Fig. 2 and 4, Figs S7 and S8). This contrasts the collective requirement for all Psa3 effectors observed here with the notion that effectors can act as public goods [25], suggesting that public compensation is not an equivalent substitute for self-sufficiency.

Many effector knockouts differed in fitness across hosts. For example, loss of the *CEL* and *hrcC* was particularly detrimental on susceptible `Hort16A', as was loss of *hopAM1a* and *hopAZ1a* (Fig. 4, Fig. S8). Similarly, *hopBN1a*, the known avirulence effector *hopF1c* [14], and *hopAU1a* appeared to be uniquely required on *A. arguta* (Fig. 4, Fig. S8). Where different hosts selected for the presence of the same effectors, susceptible `Hort16A' and resistant AA07_03 shared more overlap with each other than with tolerant `Hayward' (Fig. 4; Figs S8 and Fig. S9). These observations were supported by population structure at the final generation, where significant strain stratification was observed in a host-specific manner (Figs S8 and S9).

As observed across previous experiments, WT∆*IS* was the fittest strain (albeit, again, without statistical significance) on `Hort16A', despite several other knockout strains also increasing in the population (Fig. 4). The dominance of *ΔhopQ1a* and *ΔhopY1b* across hosts over this experiment could suggest that these were some of the few truly redundant effectors, or effectors that make the most minimal virulence contributions (Fig. 4). Sequencing of select MELEE generations suggested that these changes in population structure are driven by selection acting on the directed effector knockouts, rather than on background de novo mutations. Indeed, the only major de novo variant to emerge and increase in frequency over time was in a member of the chemotaxis gene *cheY* family, *gacA* (IYO_014595; Fig. S10). Almost every lineage had a nonsynonymous mutation emerge (ATG → AAG; M160K), the only exception being lineage 1 from `Hort16A', which instead had a high-frequency variant in another *cheY* family gene (Fig. S10). Taken together, these results revealed host-specific requirements for the majority of Psa3 effectors, supporting the emergence of Psa3 WT∆*IS* as the fittest strain in the passaged population.

### *Trans*- and partial *cis*-complementation strains of Psa3 are not fit on susceptible `Hort16A'

**Figure 5:**
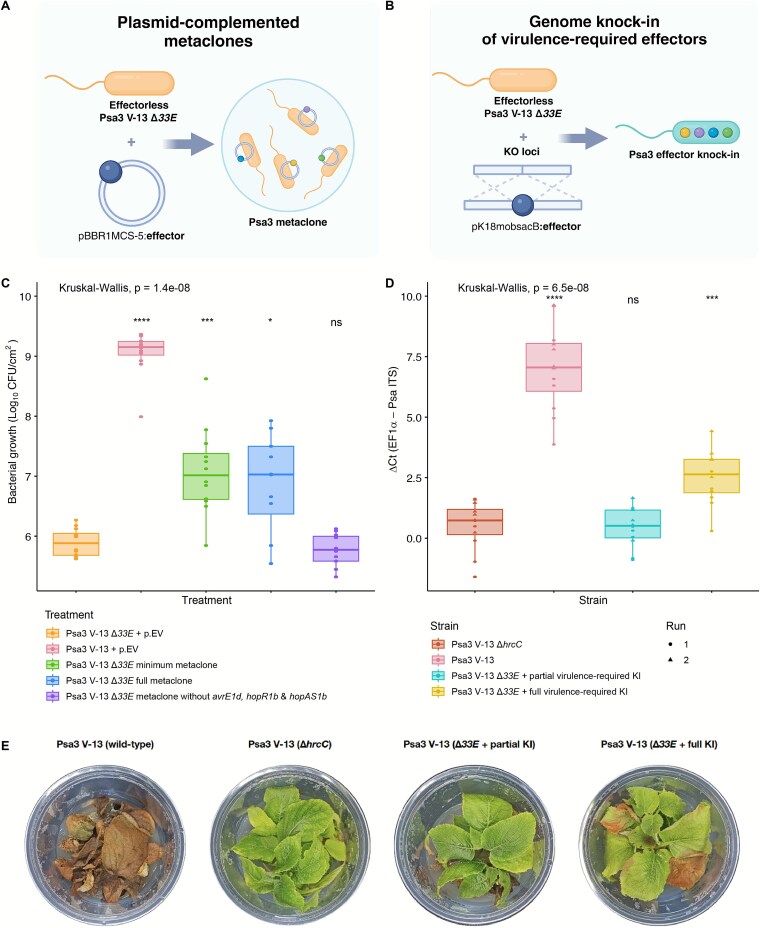
Metaclones and minimum repertoire knock-ins in an effectorless Psa3 Δ*33E* background do not achieve wild-type equivalent virulence on susceptible *A. chinensis* var. *chinensis* `Hort16A'. Schematic of (A) metaclone assembly and (B) genome knock-in methodologies. (C) Pathogenicity assay of Psa3 ∆*33E* metaclone assemblies on *A. chinensis* var. *chinensis* `Hort16A'. Bacterial growth was quantified at 12 dpi following vacuum infiltration, with three biological replicates and four pseudobiological replicates per treatment. Psa3 metaclones were constructed in an effectorless Psa3 ∆*33E* background, with each member carrying a single plasmid-based effector. The minimum metaclone was assembled from effectors expected to make considerable virulence contributions—*avrE1d, hopR1b, hopAZ1a, hopS2b, hopZ5a, hopH1a, hopAM1a, hopBP1a, hopAS1b,* and *hopI1c*. Asterisks indicate significant differences from a Wilcoxon test between the indicated strain and Psa3 ∆*33E* + p.EV (empty vector control), where *P* ≤ .05 (*), *P* ≤ .001 (***), *P* ≤ .0001 (****) and *P* ≥ .05 (ns). Thick bars represent the median values. (D) Pathogenicity assay of Psa3 ∆*33E* effector knock-in (KI) strains on *A. chinensis* var. *chinensis* `Hort16A'. Kiwifruit plantlets were flood-inoculated at ~10^6^ CFUs/ml and bacterial growth was quantified at 12 dpi by qPCR ΔCt analysis, with four pseudobiological replicates per strain. Shapes represent independent experimental runs. The Psa3 ∆*33E* + partial virulence-required KI strain has *avrE1d, hopR1b, hopAW1a, hopD2a, hopZ5a, hopH1a, hopAZ1a,* and *hopS2b* knocked in. The Psa3 ∆*33E* + full virulence-required KI strain has *hopAM1a, hopAH1, hopI1c, avrE1d, hopR1b, hopAW1a, hopD2a, hopZ5a, hopH1a, hopAZ1a, hopS2b,* and *hopBP1a* knocked in, alongside p.*hopAS1b*. Asterisks indicate significant differences from a Wilcoxon test between the indicated strain and Psa3 Δ*hrcC*, where *P* ≤ .001 (***), *P* ≤ .0001 (****) and *P* ≥ .05 (ns). Thick bars represent the median values. (E) Symptom development of Psa3 ∆*33E* effector knock-in (KI) strains on *A. chinensis* var. *chinensis* `Hort16A'. Photographs of symptom development in representative pottles were taken at 50 days post-infection.

The collective virulence of a disaggregated effector metaclone has been demonstrated in the model Pto-*Arabidopsis* pathosystem*,* suggesting that secreted effectors function as public goods [25]. However, MELEE suggests that not every effector can act as a public good, with effector loss largely selected against despite the potential for freeloading. In an attempt to recapitulate collective virulence in a different pathosystem, Psa3 metaclones (Fig. 5A) were constructed in an effectorless Psa3 ∆*33E* background [37]. Although both the full and minimum metaclones both demonstrated significantly higher *in planta* growth than the effectorless negative control, no *trans-*complemented metaclone had equivalent growth to wild-type Psa3 (Fig. 5C). Similarly, *cis*-complementing virulence required effectors by gene knock-in (Fig. 5B), either partially (5 effector loci identified in [16]) or fully (all 10 effector loci identified by MELEE), did not restore full virulence to the Psa3 ∆*33E* strain (Fig. 5D and E). The latter only partially restored virulence, strongly suggesting a significant collective role for the remaining uncomplemented effectors from Psa3.

## Discussion

Our understanding of plant-microbe interactions is often restricted by studying individual effectors or pathogens in simplified environments. This research, aligned with recent Pto DC3000-*Arabidopsis* research on cooperative virulence and effector interplay [24, 25, 61, 62], underscores the importance of examining the entire effector repertoire collectively. Serial passaging of a competitive effector knockout population confirmed known virulence requirements and identified novel contributions. Across independent lineages and replicates, WTΔ*IS* dominates *Actinidia* hosts, a phenomenon which is not observed during *in vitro* passaging nor passaging on the nonhost *M. domestica*. The fact that few effector knockouts could outcompete WT∆*IS* across *Actinidia* spp. suggests that Psa3 maintains a large effector repertoire because—despite sequence, structural, or target redundancy—almost every seemingly redundant effector makes an individual contribution to virulence. Very few of Psa3’s effectors can be lost without consequence, with their subtle contributions to virulence only apparent under the increased selective pressure of competition against co-isogenic strains. Further still, host-specific effector requirements likely provide additional selective pressure for Psa3 to retain its broad repertoire. These results offer a new lens to the paradigm that effectors are collectively essential but individually redundant. Instead, effectors in large repertoires may make subtle but cumulative contributions to virulence. This is supported by recent work in mammalian pathosystems which suggests that accessory effector repertoires, which may appear dispensable for colonisation, still have important roles in shaping infection outcomes and may contribute to overcoming barriers to infection in different tissues or permissive hosts [63, 64]. Accordingly, the existence of redundancy within a repertoire does not necessarily imply room for refinement. In contrast, generalist bacterial pathogens (e.g. *P. syringae* pv. *syringae*) and pathogens with a reduced effector complement (e.g. *P. syringae* pv. *oryzae*) demonstrate that a minimal effector repertoire may in fact be possible, albeit with some redundancy, particularly when supported by a collection of phytotoxins [65, 66].

Regardless, even in instances where full redundancy exists, or where there is a strong cost to effector carriage, there may not be an easy mechanism for effectors to be refined out of the repertoire without negatively affecting virulence. For example, Δ*hopQ1a*, Δ*hopY1b*, and Δ*hopD1a* appeared to be selected for during *in planta* passaging across both kiwifruit and apple. There is evidence in other pathosystems that HopQ1 is recognised by several resistance proteins, placing it under strong selective pressure to evade recognition—with HopR1 preventing HopQ1-elicited immunity [67]. This is particularly interesting given how strongly *hopR1b* loss appears to be selected against. Given that Δ*hopQ1a* performs well in competition, why doesn’t *hopQ1a* effector loss emerge more frequently in natural Psa3 populations? It could be that repertoire refinement is hard to achieve due to genomic linkage, with *hopQ1a* located in the exchangeable effector locus alongside other effectors with known (albeit redundant) virulence contributions [16]. There may not be an easily accessible mechanism to excise *hopQ1a* without excising these other effectors. In fact, the only observed instance of *hopQ1a* loss in the field also excised all other EEL effectors [37]. Further still, Δ*xEEL,* lacking these same effectors, is not competitively fit during passaging. Indeed, using these block mutants which delete co-located effectors may actually better mimic the dynamics of gene loss and gain observed in the field than individual knockouts. When considered alongside the dearth of effector mutation and repertoire refinement in orchard populations [14, 37, 38], these observations, across different time scales and settings, reframe our understanding of the redundant majority of effectors in plant pathogen repertoires. Specifically, the competitive fitness of WTΔ*IS* suggests that HopQ1a, and all other recognised effectors, still provides useful virulence functions that another effector cannot provide redundantly.

A curious discovery from the two experimental kiwifruit passaging setups (3-generation first run versus 9-generation second run) was the observation of differential `selection pressures' and their impact on the knockout strain drop-outs between the two runs. The second run appeared to be a more `gentle' selection for strain fitness—this was demonstrated by the lack of phenomena observed in 3-generation run: WTΔ*IS* prominence rising rapidly; Δ*fEEL*/Δ*sEEL*/Δ*hopF1c* strains occasionally falling out of the population in at least one replicate; and, Δ*hopR1b* dropping out to an undetectable level. It is unclear why the two runs differed in this way, but may involve pre-existing subtle founder effects from the inoculum populations or seasonal effects of laboratory setup.

Metaclone systems in Pto and *P. syringae* pv. *syringae* have demonstrated that virulence can be constructed from many parts, with strains carrying and sharing different virulence components [21, 25]. However, MELEE demonstrated that the loss of a single virulence component in a given strain often weakened its virulence, despite being surrounded by other strains carrying that component. Furthermore, we were unable to reconstitute a fully virulent Psa3 metaclone. It is worth noting that differences between our implementation of a metaclone system and the original Pto system [25], which could alternatively explain our different findings. Firstly, plants were flood inoculated with a Psa3 metaclone at an OD of 0.005, compared to spray inoculation at an OD of 2 (or syringe infiltration at a lower OD). Psa3 effectors on pBBR1MCS5 plasmids were expressed under a highly expressed *avrRps4* promoter, rather than under their native promoter, which may alter the stoichiometry between effectors and thus affect the roles each effector plays in its otherwise native context. Finally, the Psa-kiwifruit pathosystem lacks the same clear identification of immune-eliciting effectors as the Pto-*Arabidopsis* pathosystem, hence these could not be comprehensively removed from the Psa3 V-13 metaclones. Nevertheless, this work challenges the notion that all effectors can act as public goods, suggesting that some effectors may be private goods and that, even when public, trans-complementation cannot provide equivalent fitness in a community context compared to self-sufficiency.

Differences in experimental systems may also explain the disparities observed – in particular, bacterial load. Cross-complementation within these populations may only be possible at unnaturally high bacterial densities. Bacterial populations delivered by syringe or vacuum infiltration, particularly at high concentrations, are artificial and may not mimic natural infection contexts [12, 18, 21, 23–25, 62, 68–73]. In contrast, topical application through spraying, dipping, drenching or flooding better reflects natural infection mediated through the water cycle [15, 74–79]. These methodological choices are especially critical in the context of community assemblies, as public goods production and sharing are known to be density-dependent [80]. As demonstrated in *Yersinia pestis*, an avirulent strain’s proximity to a virulent strain can determine its success [28]. Fullmer *et al.* [81] have recently posited that interaction range—the number of beneficiaries a producer can support—shapes public goods sharing dynamics. Disaggregated effector repertoires in metaclonal populations represent a maximally distributed system. Conversely, in this research, the initial intermediate state of MELEE progresses to be dominated by the maximally centralised WT∆*IS.* The interaction range and population density of a system influence neighbour uncertainty, which can harm nonproducers if they become spatially separated from and cannot reliably interact with producers [81, 82]. At lower population densities, this uncertainty promotes self-sufficiency over disaggregation and interdependence [82]. Furthermore, we expect that the inhospitable environment of the plant apoplast would further contract the interaction range. This has several consequences when considering the work at hand alongside previous on-orchard and *in vitro* research [14–16, 37, 38, 60, 83]. Firstly, the initial population density and infiltration method may strongly influence the dynamics observed. Secondly, knockouts (or mutants) of effectors involved in the early stages of host entry and colonisation may be under stronger selection, as strains may be more spatially isolated in a small initial population, limiting the opportunity for public goods to be shared. Thus, although metaclone assembly is a useful tool to demonstrate the cooperative performance of effectors, in its current form it is not necessarily representative of natural infection dynamics [25]. These works may, therefore, fail to capture important subtleties, much like the limitations observed for traditional single-isolate pathogenicity assays.

This research has also highlighted questions around the potential redundancy and specialisation of Psa’s large β-barrel effectors that are critical for virulence [16]. There is a separation between Δ*CEL* (which has lost *avrE1d*) and Δ*hopR1b,* which suggests that these effector knockouts are selected against to different degrees. There appears to be a gradient of requirement for these β-barrel effectors, with HopR1b loss strongly selected against, then AvrE1d, and finally HopAS1b, the loss of which is only weakly selected against in `Hort16A' and AA07_03. Could it be that although AvrE1d is recognised, HopR1b is not and, therefore, there is no benefit to HopR1b loss? Or could it be that despite a similar structure, these effectors have specialised functions throughout infection? If HopR1b has a role in early host entry through stomata [60], individual cells may be more isolated at the outset of infection and, therefore, less able to share goods. When we consider public goods or costs, the spatiotemporal dynamics of infection must be considered for a nuanced understanding of redundancy potential. Furthermore, Nomura *et al.* [84] show that AvrE acts as a water channel and also allows the passage of small molecules. If these channels aided the movement of effectors, toxins, or other small molecules, either out of the cell or into organelles, this could explain the differing requirements for AvrE1/HopR1 that have emerged across Pto and Psa [15, 18, 85]. A natural progression of this research would be to study the prevalence and redundancy of β-barrel effectors, which seem to be so central for plant pathogenesis, across diverse plant pathogens.

There may also be an evolutionary benefit in retaining redundant, secreted effectors. There are several mechanisms through which new gene functions can emerge, with gene duplication often considered one of the primary mechanisms allowing neofunctionalisation [86]. More recently, a constructive black queen hypothesis has been proposed [87]. The classical black queen hypothesis puts forth that `leaky' common goods may lead to adaptive gene loss and reductive genome evolution, as the loss of costly leaky function is selected for in individual strains, so long as it is retained at the community level [88]. The constructive black queen hypothesis suggests that public goods create redundancy, buffering the consequences of genetic loss of function [87]. Ultimately, this redundancy facilitates the emergence of novel genetic diversity and gain of function in the long-term [87]. Therefore, product sharing may accelerate the evolution of gene neofunctionalisation. If public goods sharing allows faster evolution through genetic redundancy, does the redundant portion of a repertoire, by its very nature, facilitate the adaptive potential of a pathogen, allowing adaptation to host immunity and, potentially, host jumps? This could explain why collective requirement emerges from apparent redundancy if redundant effectors have unique off-target or moonlighting functions. Although we tend to focus on nonredundant effectors that make essential contributions to virulence when considering resistance breeding strategies, the potential for redundant effectors to generate diversity is worth considering as a resistance-breaking strategy.

Understanding how pathogens emerge, evolve and cause disease is crucial to protect hosts during disease outbreaks. Critically, we must advance our understanding of the molecular mechanisms underlying pathogenicity, effector, and community interactions, whether this be for individual pathogens or collectively virulent communities [89–91]. MELEE has allowed us to explore weaker virulence contributions, thereby offering a distinct approach that better mimics natural infection cycles and reveals more about intra-strain effector function. Future research should attempt to transition these more artificial infection mechanisms towards those that more fully replicate natural dynamics, to better ensure that findings are relevant to real populations, microbial communities, and environments. Ultimately, effectors alone are not virulent—strains are. If collective virulence or tolerance of repertoire perturbation only occurs in particular environments, this could be discerned by assembling different repertoires, with different degrees of disaggregation, through effector knockouts, knock-ins, and metaclone assembly. A more detailed understanding of effector-mediated virulence as a public good and how repertoires aggregate and disaggregate over time will help us better understand pathogen emergence and the wider evolution of virulence across pathosystems.

## Supplementary Material

ISME_Supplemental_material_wraf262

## Data Availability

All analysed data from this study are included in this published article (and its supplementary information file). The datasets generated during and/or analysed during the current study are available in the Zenodo repository (https://zenodo.org/records/17675153).
