# Supplementary material for: Individually redundant effectors are collectively required for bacterial pathogen virulence
Source: ISME J. 2025 Nov 26;19(1):wraf262. doi: 10.1093/ismejo/wraf262 (PMC12694402; doi:10.1093/ismejo/wraf262)
Supplement: ISME_Supplemental_material_wraf262 [file isme_supplemental_material_wraf262.docx]

**Supporting information**

**
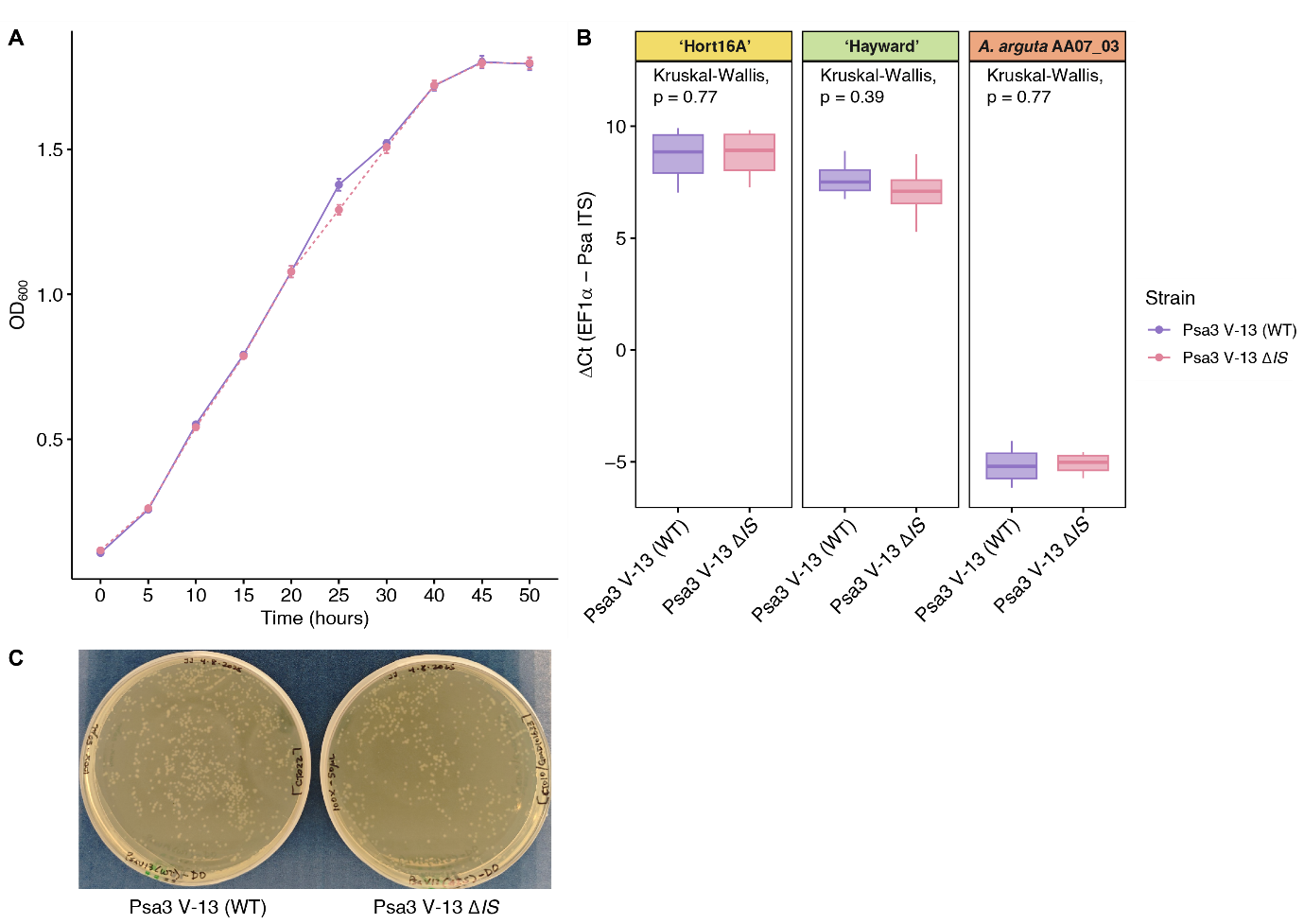
**

**Supplementary Figure S1. *In vitro* and *in planta* growth of the wild-type-like strain Psa3 V-13 Δ*IS*.** (A) *In vitro* growth assay of Psa3 V-13 strains. Points represents the mean OD_600_ and error bars represent the standard error of the mean (SEM) for six independent replicates. (B) Pathogenicity assay of Psa3 V-13 strains on *Actinidia chinensis* var. *chinensis* ‘Hort16A’, *A. chinensis* var. *deliciosa* ‘Hayward’, and *A. arguta* AA07_03. Tissue culture plantlets were flood-inoculated at approximately 10^6^ CFUs/mL. Bacterial growth was quantified at 12 days post-inoculation by qPCR ΔCt analysis. with the bars representing the median values and whiskers representing the 1.5 inter-quartile range for four pseudobiological replicates per strain. (C) Growth of Psa3 V-13 strains incubated on LB agar supplemented with nitrofurantoin and cephalexin.


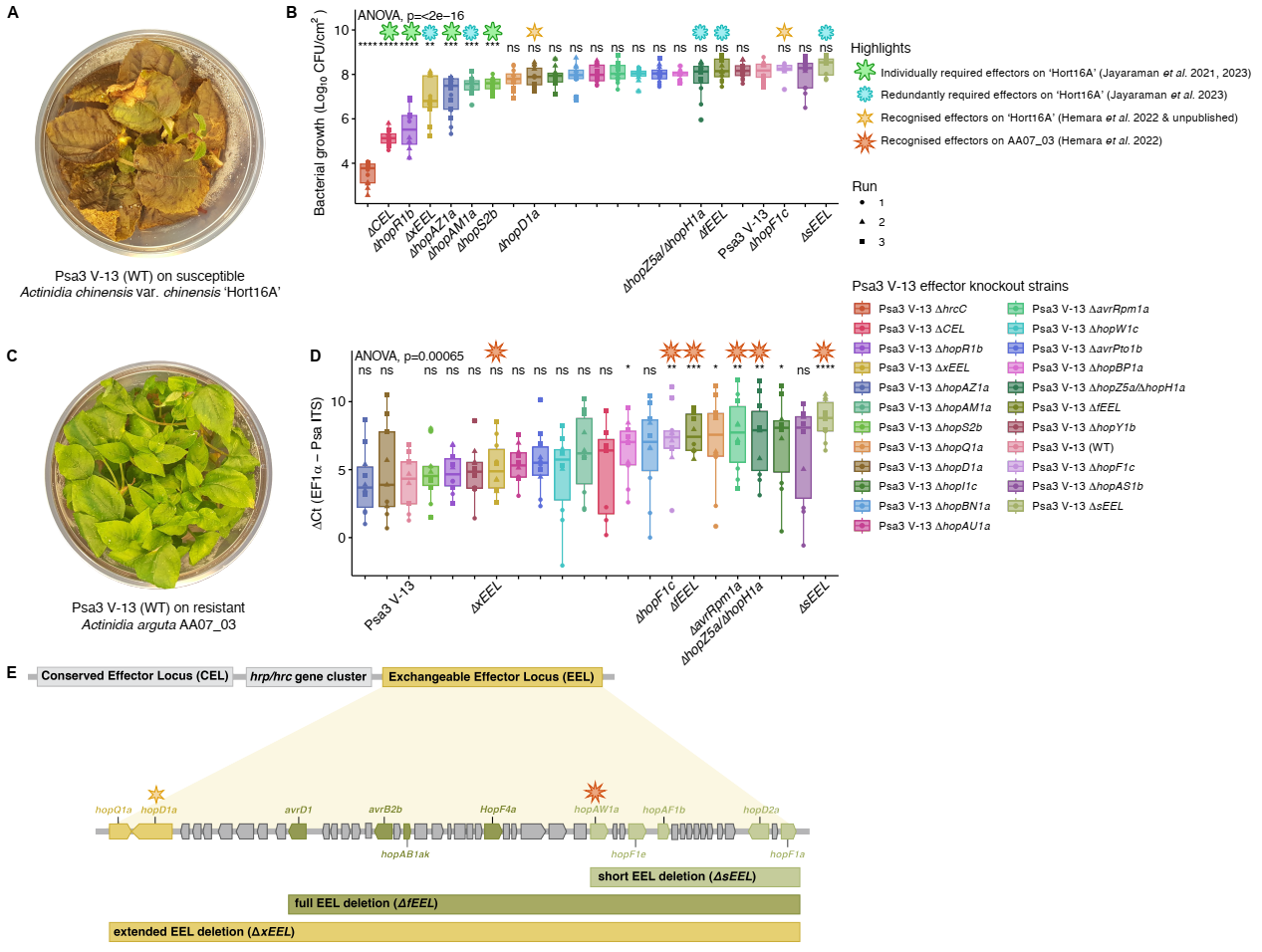


**Supplementary Figure S2. Summary of Psa3 V-13 effector knockout research to date.** (A) Symptom development of Psa3 V-13 (WT) on *Actinidia chinensis* var. *chinensis* ‘Hort16A’. ‘Hort16A’ kiwifruit plantlets were flood-inoculated at approximately 10^7^ CFUs/mL. Photographs of symptom development in representative pottles were taken at 50 days post-infection. (B) Psa3 effectors required for virulence. *Actinidia chinensis* var. *chinensis* ‘Hort16A’ plantlets were flood inoculated with wild-type Psa3 V-13, Δ*hrcC*, and effector knockout strains at approximately 10^6^ CFU/mL. Bacterial growth was quantified at 12 days post-inoculation (dpi) by serial dilution and plate-count quantification. *in planta* bacterial counts were plotted as Log_10_ CFU/cm^2^, with thick bars representing the median values and whiskers representing the 1.5× interquartile range from four pseudobiological replicates across three independent experimental runs. Asterisks indicate significant differences from Welch's t-test between the indicated strain and WT Psa3: *, P ≤ 0.05; **, P ≤ 0.01; ***, P ≤ 0.001; ****, P ≤ 0.0001; ns, not significant. Green seven-pointed stars represent effectors individually required for virulence (Jayaraman *et al.* 2021, 2023); blue ten-pointed stars represent effectors redundantly required for virulence on ‘Hort16A’ (Jayaraman *et al.* 2023). Yellow six-pointed stars represent effectors recognised on ‘Hort16A’ (Hemara *et al.* 2022 & unpublished). (C) Symptom development of Psa3 V-13 (WT) on *Actinidia arguta*. *A. arguta* AA07_03 kiwifruit plantlets were flood-inoculated at approximately 10^7^ CFUs/mL. Photographs of symptom development in representative pottles were taken at 50 days post-infection. (D) Pathogenicity assay screen of Psa3 V-13 effector knockout strains in *Actinidia arguta* identifies four avirulence loci. Reproduced from Hemara *et al.* (2022). *A. arguta* AA07_03 kiwifruit plantlets were flood-inoculated at approximately 10^6^ CFUs/mL. Psa biomass (ITS) was quantified relative to AaEF1α using the ΔCt analysis method for three pseudobiological replicates, per strain, per experimental run. Box and whisker plots, with thick bars representing the median values and whiskers representing the 1.5 inter-quartile range. Asterisks indicate the statistically significant difference of Student’s t-test following ANOVA between the indicated strain and wild-type Psa3 V-13, where p ≤.05 (*), p≤.01 (**), p≤.001 (***), p≤.0001 (****), and p>.05 (ns; not significant). This experiment was separately conducted three times (biological replicates) with three batches of independently grown plants and data were stacked to generate the box plots. Red nine-pointed stars represent effectors recognised by AA07_03 (Hemara *et al.* 2022). (E) Schematic of the effectors comprising the exchangeable effector locus (EEL), encompassing the extended EEL (xEEL; *hopQ1a* –*hopF1a*), full EEL (fEEL; *avrD1* –*hopF1a*), and short EEL (sEEL; *hopAW1a* –*hopF1a*) loci in Psa3 V-13. The yellow six-pointed star and the red nine-pointed star represent effectors recognised on ‘Hort16A’ and AA07_03, respectively (Hemara *et al.* 2022).

**
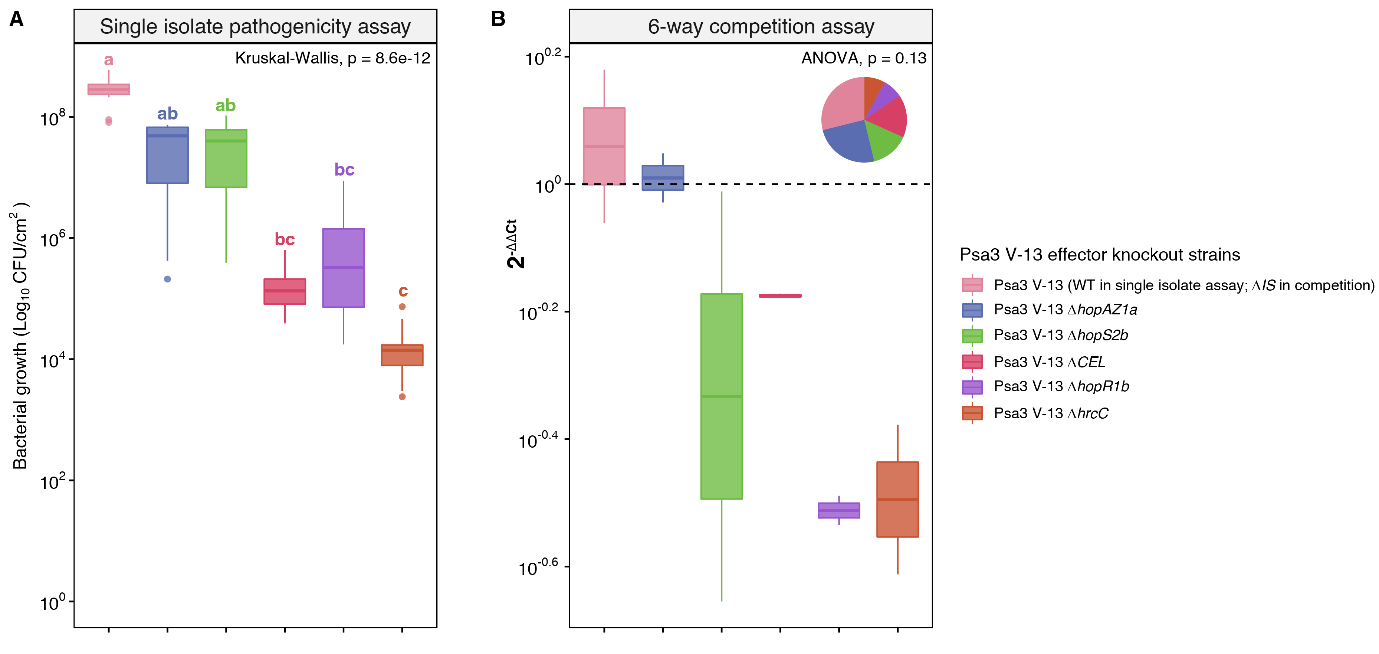
**

**Supplementary Figure S3. Psa3 virulence-required effector knockout strains perform differently across single isolate and competitive fitness assays on susceptible *A. chinensis* var. *chinensis* ‘Hort16A’**. (A) Bacterial growth was quantified at 12 days post-inoculation using plate count quantification. Effector knockout strains with different letters are significantly different at α ≤ 0.05, as determined by Nemenyi's non-parametric all-pairs comparison test. (B) Six-way competition of Psa3 ∆*IS*, ∆*hopAZ1a,* ∆*hopS2b,* ∆*CEL,* ∆*hopR1b,* and ∆*hrcC* at 12 days post-inoculation. The dashed line represents a 2^-∆∆Ct^ value of 1. Strains above this line have increased relative to the starting population, whereas strains below this line have decreased. The pie charts show the mean 2^-∆∆Ct^ value for each effector knockout strain.

**
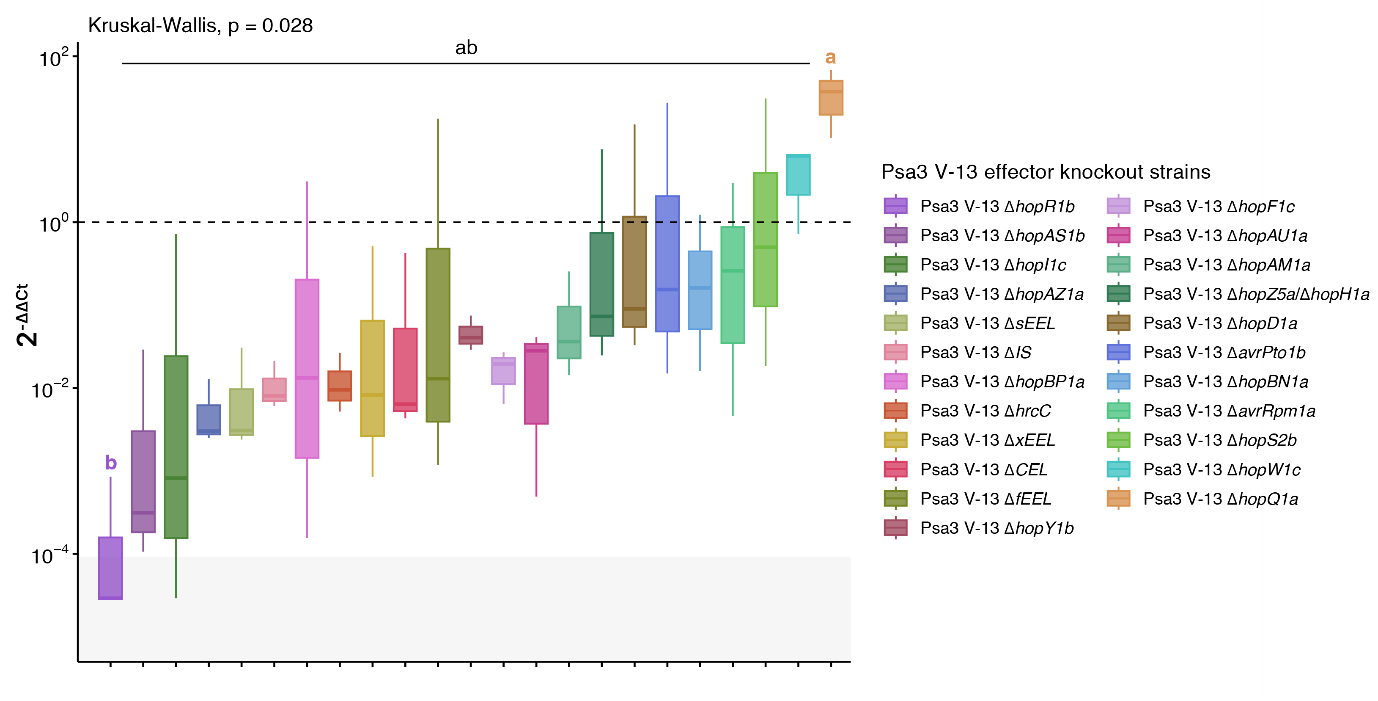
**

**Supplementary Figure S4. Generation three of serial passaging a competitive Psa3 effector knockout population on *Malus domestica* ‘Royal Gala’ tissue culture plantlets.** The dashed line represents a 2^-∆∆Ct^ value of 1. Strains above this line have increased relative to the starting population, whereas strains below this line have decreased. The grey box highlights the range of 2^-∆∆Ct^ values that correspond to raw Ct values of 40, indicating that the effector knockout strain could not be detected by qPCR. The boxplots show the relative abundance of effector knockout strains over time, normalised to Psa ITS for each generation and the starting population. Effector knockout strains with different letters are significantly different at α ≤ 0.05, as determined by Nemenyi's non-parametric all-pairs comparison test.

**
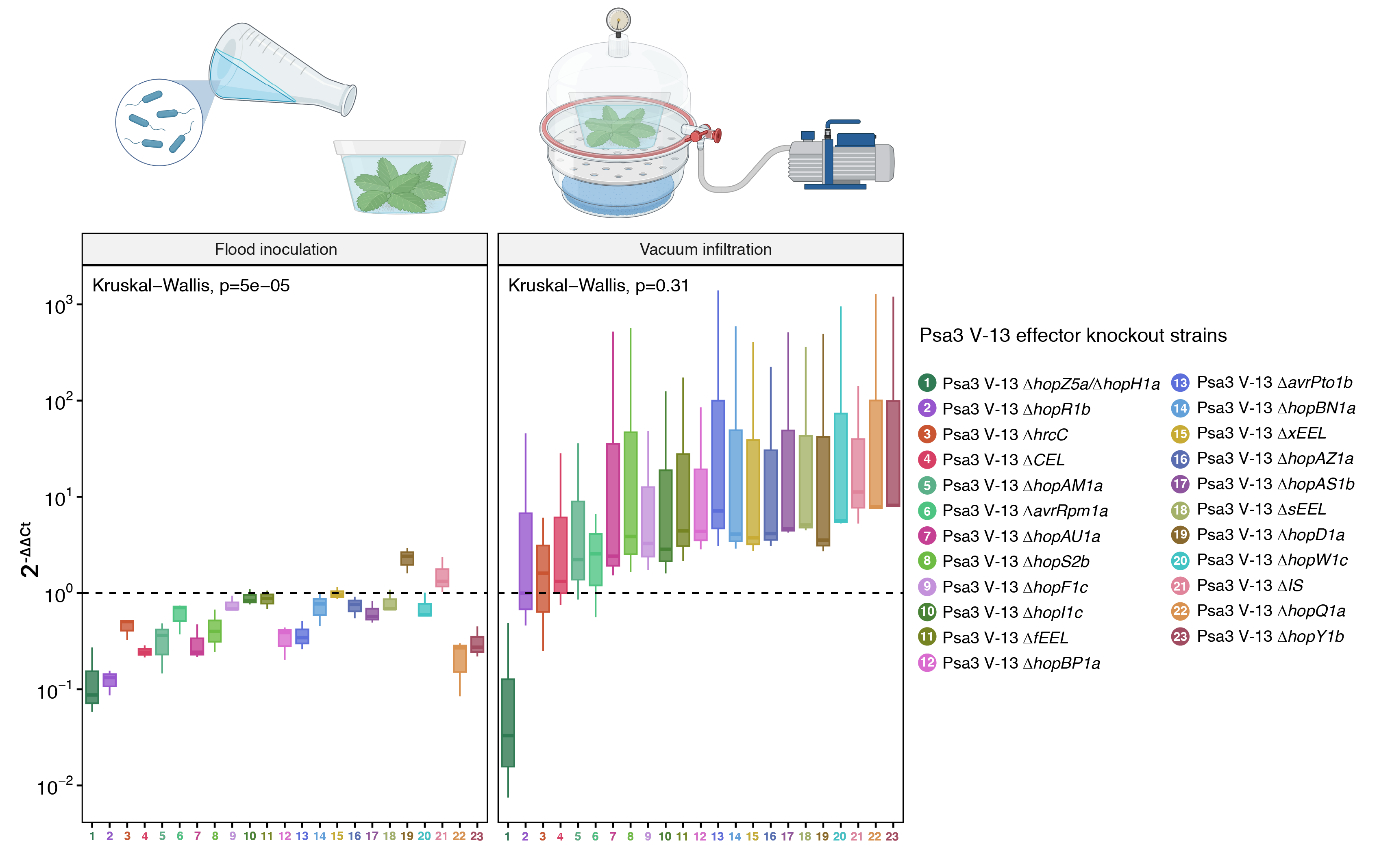
**

**Supplementary Figure S5. Vacuum infiltration of a competitive Psa3 V-13 effector knockout population on *Actinidia chinensis* var. *chinensis* ‘Hort16A’.** Strain abundance following vacuum infiltration was quantified at 12 days post-inoculation (dpi) by qPCR.The dashed line represents a 2^-∆∆Ct^ value of 1. Strains above this line have increased relative to the starting population, while strains below this line have decreased. The flood inoculation panel depicts data from generation 1 of serial passaging on ‘Hort16A’ (Figure 2).

**
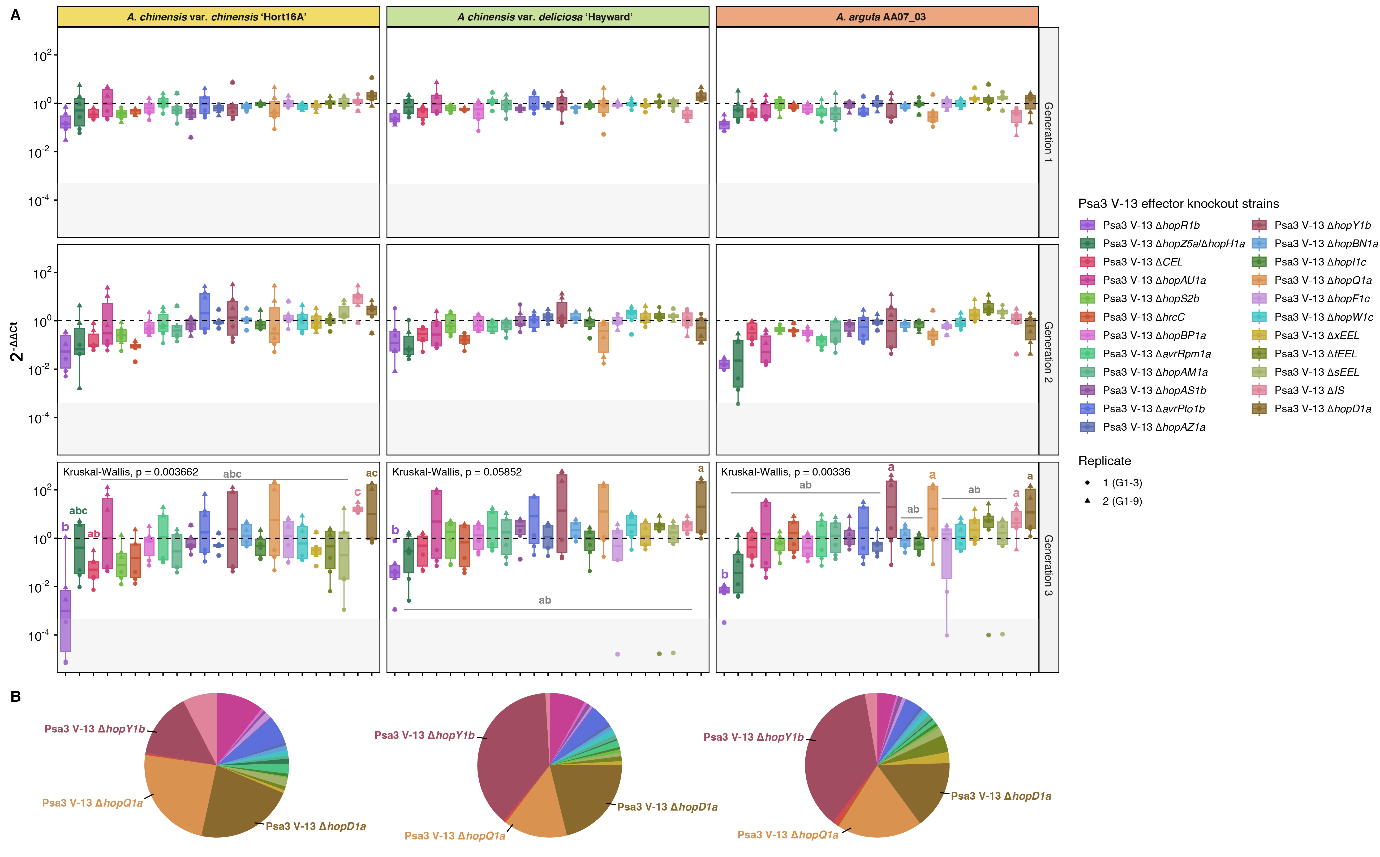
**

**Supplementary Figure S6. Comparing two independent serial passaging experiments of a competitive Psa3 effector knockout population across *A. chinensis* var. *chinensis* ‘Hort16A’, *A. chinensis* var. *deliciosa* ‘Hayward’, and *A. arguta* tissue culture plantlets. (**A) The dashed line represents a 2^-∆∆Ct^ value of 1. Strains above this line have increased relative to the starting population, whereas strains below this line have decreased. The grey box highlights the range of 2^-∆∆Ct^ values that correspond to raw Ct values of 40, indicating that the effector knockout strain could not be detected by qPCR. The boxplots show the relative abundance of effector knockout strains over time, normalised to Psa ITS for each generation, and to the starting population. The point shape indicates the experimental run, with passage 1 (G1-3) represented by circles and passage 2 (G1-9) represented by triangles. For generation 3, effector knockout strains with different letters are significantly different at α ≤ 0.05, as determined by Nemenyi's non-parametric all-pairs comparison test. (B) The pie charts show the mean 2^-∆∆Ct^ value at generation 3 for each effector knockout strain for *A. chinensis* var. *chinensis* ‘Hort16A’, *A. chinensis* var. *deliciosa* ‘Hayward’, and *A. arguta* AA07_03.

**
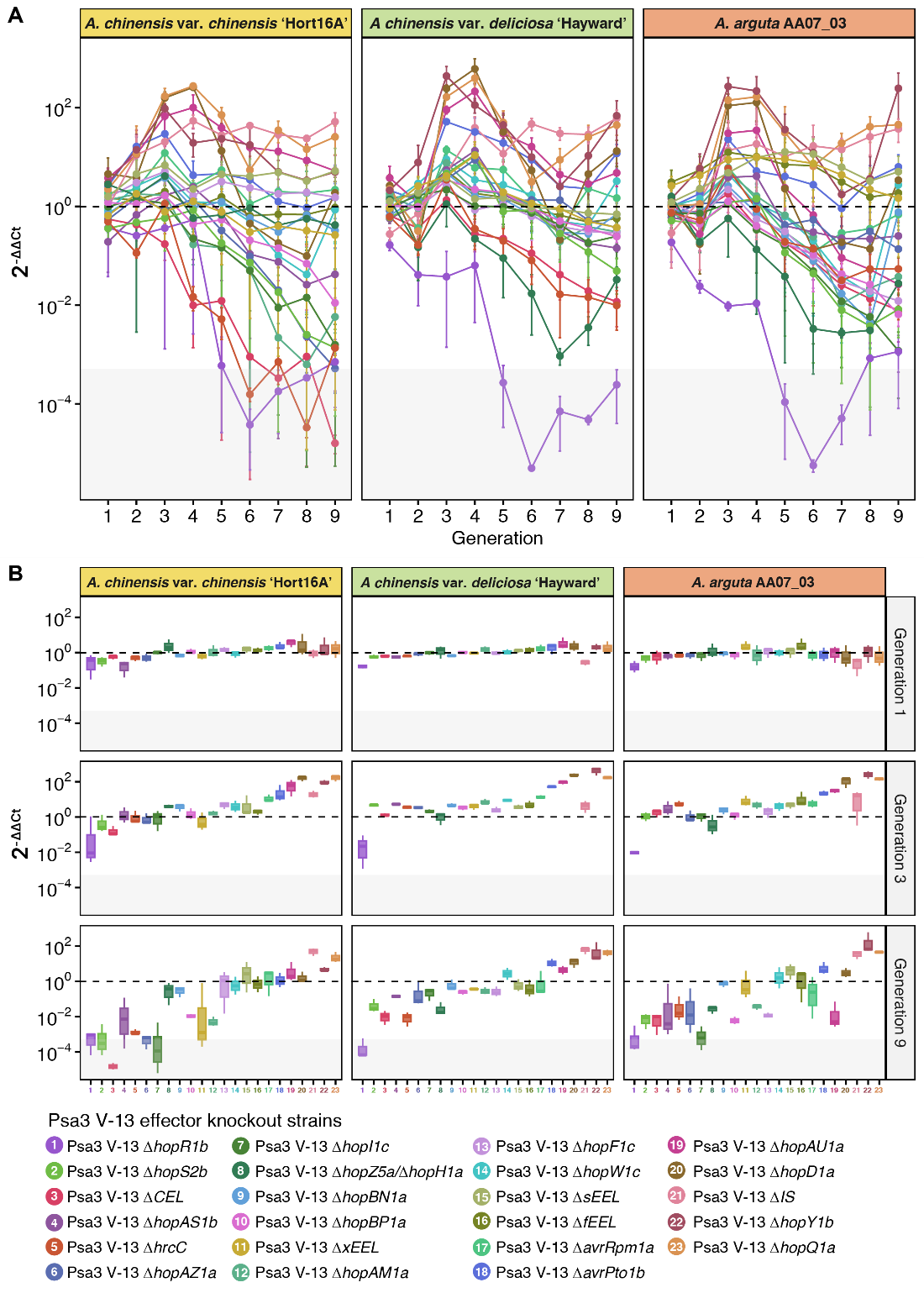
**

**Supplementary Figure S7. Nine generations of serial passaging a competitive Psa3 effector knockout population across *A. chinensis* var. *chinensis* ‘Hort16A’, *A. chinensis* var. *deliciosa* ‘Hayward’, and *A. arguta* AA07_03 tissue culture plantlets.** The dashed line represents a 2^-∆∆Ct^ value of 1. Strains above this line have increased relative to the starting population, whereas strains below this line have decreased. The grey box highlights the range of 2^-∆∆Ct^ values that correspond to raw Ct values of 40, indicating that the effector knockout strain could not be detected by qPCR. (A) The line graph shows the relative abundance of effector knockout strains over time, normalised to Psa ITS for each generation, and to the starting population. The round point represents the mean and error bars represent standard error across three replicate lineages. (B) The boxplots show the relative abundance of effector knockout strains over time at the key generations 1, 3, and 9, normalised to Psa ITS for each generation, and to the starting population.

**
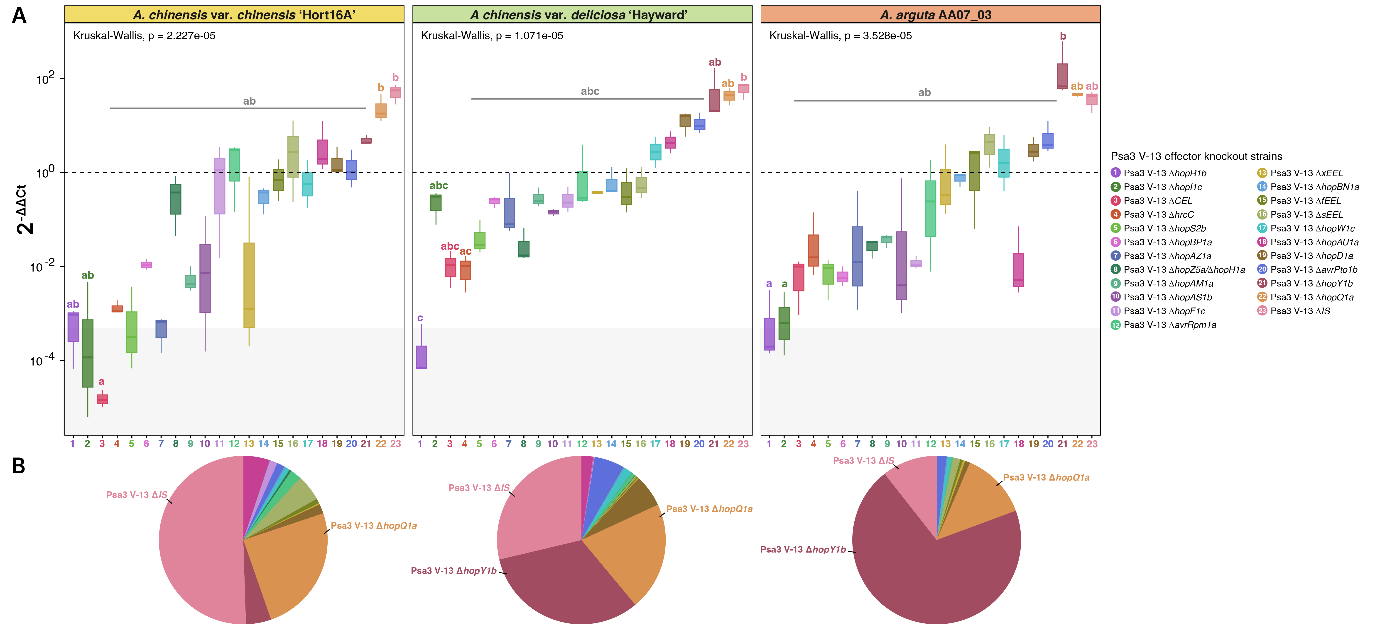
**

**Supplementary Figure S8. Generation nine of serial passaging a competitive Psa3 effector knockout population on *A. chinensis* var. *chinensis* ‘Hort16A’, *A. chinensis* var. *deliciosa* ‘Hayward, and *Actinidia arguta* AA07_03 tissue culture plantlets.** (A) The dashed line represents a 2^-∆∆Ct^ value of 1. Strains above this line have increased relative to the starting population, whereas strains below this line have decreased. The grey box highlights the range of 2^-∆∆Ct^ values that correspond to raw Ct values of 40, indicating that the effector knockout strain could not be detected by qPCR. The boxplots show the relative abundance of effector knockout strains over time, normalised to Psa ITS for each generation and the starting population. Effector knockout strains with different letters are significantly different at α ≤ 0.05, as determined by Nemenyi's non-parametric all-pairs comparison test. (B) The pie charts show the mean 2^-∆∆Ct^ value at generation 9 for each effector knockout strain for *A. chinensis* var. *chinensis* ‘Hort16A’, *A. chinensis* var. *deliciosa* ‘Hayward’, and *A. arguta* AA07_03.


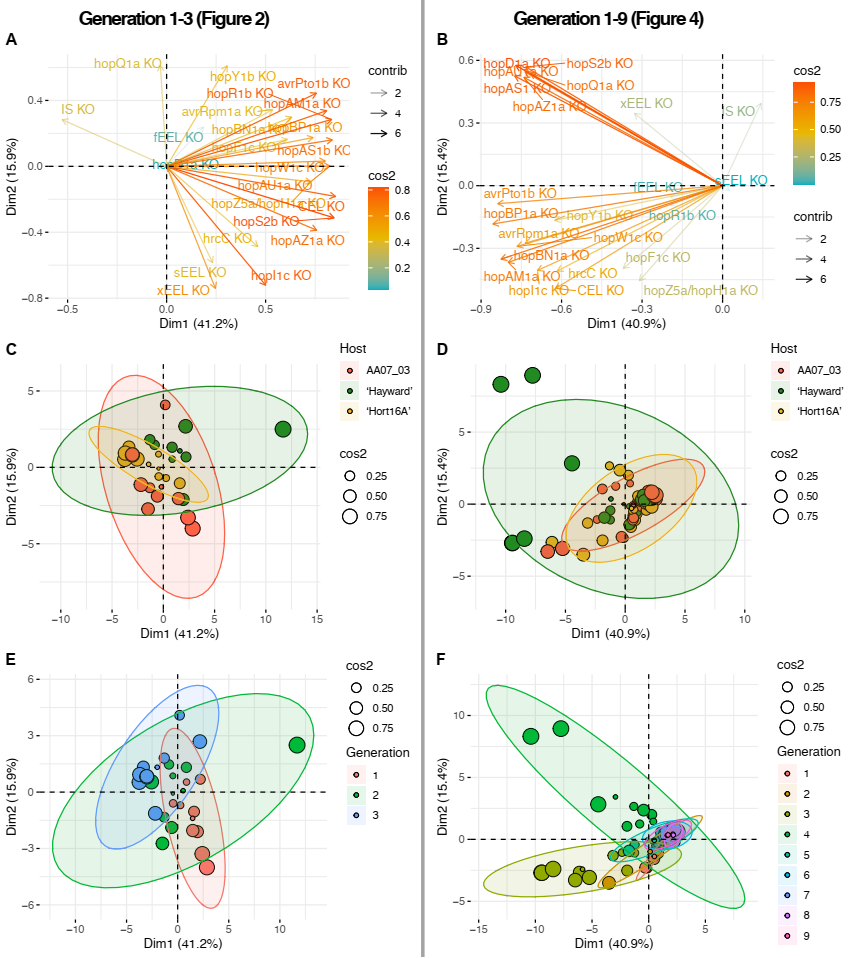


**Supplementary Figure S9. Principal components analysis (PCA) of three generations (A, C, E) and nine generations (B, D, F) of serial passaging data.** (A-B) PCA biplot grouped by effector knockout strain. Each strain is coloured by cos2, which indicates how much of a variable’s variance is represented by the principal components. (C-D) Individual points on a PCA plot, where point size indicates cos2 representation, and point and ellipse colour represents the host. (E-F) Individual points on a PCA plot, where point size indicates cos2 representation, and point and ellipse colour indicates the passaging generation.


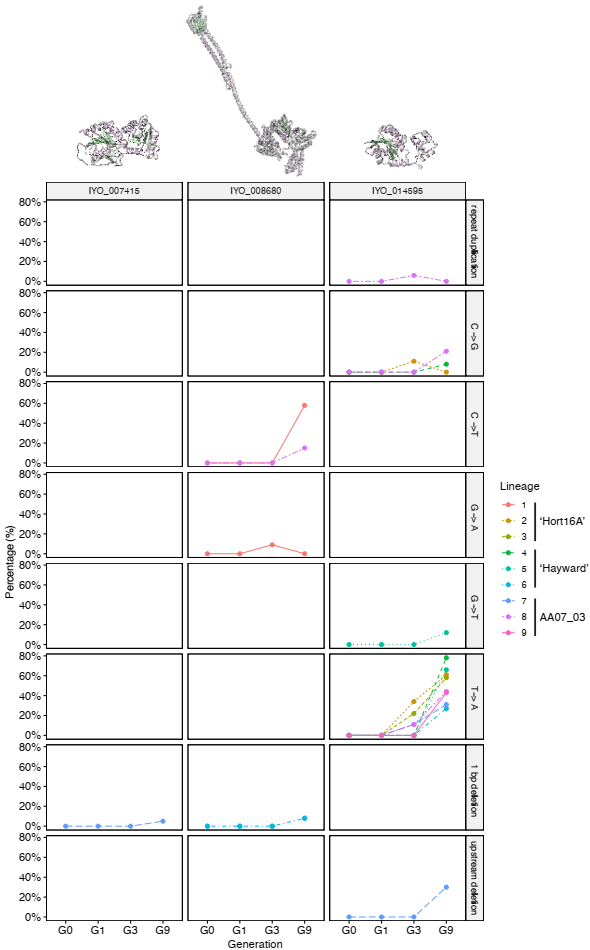


**Supplementary Figure S10. *cheY* variants have emerged in Psa3 effector knockout serial passaging populations at high frequencies across all hosts and lineages.** Breseq ([Barrick *et al.* 2014)](https://www.zotero.org/google-docs/?fnC0zI) (version 0.38.1) was used to call variants from short-read sequencing of the initial inoculum (i.e. G0) and generations one, three and nine, relative to Psa3 V-13. Host and lineage of origin are indicated. Predicted protein structures for each *cheY* gene of interest (IYO_007415, IYO_008680, IYO_014595) are displayed.

**Supplementary Table 1. Psa3 V-13 effector knockout strains and Psa3 V-13 ∆*33E* plasmid-complemented strains used in this study.**

| **Strain** | **Description** | **Source** |
| --- | --- | --- |
| Psa3 V-13 ∆*IS* | Deleted the insertion sequence IYO_001195 (A.K.A. IS285) | [(Hemara *et al.* 2025b)](https://www.zotero.org/google-docs/?e93Q5p) |
| Psa3 V-13 ∆*hrcC* | deleted *hrcC* | [(Straub *et al.* 2018)](https://www.zotero.org/google-docs/?Ka1BlW) |
| Psa3 V-13 ∆*sEEL* | deleted *hopAW1a, hopF1e* (and associated *shcF*)*, hopD2a, hopAF1b,* and *hopF1a (*and associated *shcF*) | [(Hemara *et al.* 2022)](https://www.zotero.org/google-docs/?HV8MTe) |
| Psa3 V-13 ∆*fEEL* | deleted *avrD, hopF4a (*and associated *shcF*)*, avrB2b, hopAW1a, hopF1e (*and associated *shcF*)*, hopD2a, hopAF1b, and hopF1e (*and associated *shcF*) | [(Hemara *et al.* 2022)](https://www.zotero.org/google-docs/?YXzLsS) |
| Psa3 V-13 ∆*xEEL* | *deleted hopQ1a, hopD1a, avrD, hopF4a (*and associated *shcF*)*, avrB2b, hopAW1a, hopF1e (*and associated *shcF*)*, hopD2a, hopAF1b,* and *hopF1a (*and associated *shcF*) | [(Hemara *et al.* 2022)](https://www.zotero.org/google-docs/?GauRui) |
| Psa3 V-13 ∆*CEL* | deleted *hopN1a, shcM*, *hopM1f, hrpW1, shcE* and *avrE1d* | [(Jayaraman *et al.* 2020)](https://www.zotero.org/google-docs/?uWgH0d) |
| Psa3 V-13 ∆*hopZ5a*/∆*hopH1a* | deleted *hopZ5a* and *hopH1a* | [(Hemara *et al.* 2022)](https://www.zotero.org/google-docs/?opiS5X) |
| Psa3 V-13 ∆*hopAM1a-1*/∆*hopAM1a-2* | deleted *hopAM1a-1* and *hopAM1a-2*; (two separate loci) | [(Hemara *et al.* 2022)](https://www.zotero.org/google-docs/?jMhm3l) |
| Psa3 V-13 ∆*hopQ1a* | deleted *hopQ1a* | [(Hemara *et al.* 2022)](https://www.zotero.org/google-docs/?asCqvW) |
| Psa3 V-13 ∆*hopD1a* | deleted *hopD1a* | [(Hemara *et al.* 2022)](https://www.zotero.org/google-docs/?VPd29d) |
| Psa3 V-13 ∆*hopI1c* | deleted *hopI1c* | [(Hemara *et al.* 2022)](https://www.zotero.org/google-docs/?naaWCA) |
| Psa3 V-13 ∆*hopY1b* | deleted *hopY1b* | [(Hemara *et al.* 2022)](https://www.zotero.org/google-docs/?1CYKBr) |
| Psa3 V-13 ∆*avrRpm1a* | deleted *avrRpm1a* | [(Hemara *et al.* 2022)](https://www.zotero.org/google-docs/?3BFAQa) |
| Psa3 V-13 ∆*hopW1c* | Deleted *hopW1c* | [(Hemara *et al.* 2022)](https://www.zotero.org/google-docs/?tXYXkP) |
| Psa3 V-13 ∆*hopBN1a* | Deleted *hopBN1a* | [(Hemara *et al.* 2022)](https://www.zotero.org/google-docs/?InW8ml) |
| Psa3 V-13 ∆*hopAZ1a* | Deleted *hopAZ1a* | [(Hemara *et al.* 2022)](https://www.zotero.org/google-docs/?mazPFL) |
| Psa3 V-13 ∆*hopR1b* | deleted *hopR1b* (extended region owing to flanking repeat sequences) | [(Jayaraman *et al.* 2020)](https://www.zotero.org/google-docs/?sDa7Ax) |
| Psa3 V-13 ∆*hopF1c* | Deleted *hopF1c* | [(Hemara *et al.* 2022)](https://www.zotero.org/google-docs/?MRTS5a) |
| Psa3 V-13 ∆*hopAU1a* | Deleted *hopAU1a* | [(Hemara *et al.* 2022)](https://www.zotero.org/google-docs/?DfjXw9) |
| Psa3 V-13 ∆*hopBP1a* | Deleted *hopBP1a* | [(Hemara *et al.* 2022)](https://www.zotero.org/google-docs/?cD8VFT) |
| Psa3 V-13 ∆*hopAS1b* | Deleted *hopAS1b* | [(Hemara *et al.* 2022)](https://www.zotero.org/google-docs/?YdTAVu) |
| Psa3 V-13 ∆*avrPto1b* | Deleted *avrPto1b* | [(Hemara *et al.* 2022)](https://www.zotero.org/google-docs/?jtscxk) |
| Psa3 V-13 ∆*hopS2b* | Deleted *hopS2b* | [(Hemara *et al.* 2022)](https://www.zotero.org/google-docs/?0T0ohE) |
| Psa3 V-13 Δ33E + pBBR1MCS-5 (EV) | Plasmid-complemented with empty vector (pBBR1MCS-5B) | [(Hemara *et al.* 2025a)](https://www.zotero.org/google-docs/?VHaaub) |
| Psa3 V-13 Δ33E + pBBR1MCS-5:*avrB2b* | Plasmid-complemented with *avrB2b* (cloned under the *avrRps4* promoter) | This study. |
| Psa3 V-13 Δ33E + pBBR1MCS-5:*avrD1a* | Plasmid-complemented with *avrD1a (*cloned under the *avrRps4* promoter) | This study. |
| Psa3 V-13 Δ33E + pBBR1MCS-5:*avrE1d* | Plasmid-complemented with *avrE1d (*cloned under the *avrRps4* promoter) | This study. |
| Psa3 V-13 Δ33E + pBBR1MCS-5:*avrPto1b* | Plasmid-complemented with *avrPto1b (*cloned under the *avrRps4* promoter) | This study. |
| Psa3 V-13 Δ33E + pBBR1MCS-5:*avrRpm1a* | Plasmid-complemented with *avrRpm1a (*cloned under the *avrRps4* promoter) | This study. |
| Psa3 V-13 Δ33E + pBBR1MCS-5:*hopW1c* | Plasmid-complemented with *hopW1c (*cloned under the *avrRps4* promoter) | This study. |
| Psa3 V-13 Δ33E + pBBR1MCS-5:*hopAF1b* | Plasmid-complemented with *hopAF1b (*cloned under the *avrRps4* promoter) | This study. |
| Psa3 V-13 Δ33E + pBBR1MCS-5:*hopAH1a* | Plasmid-complemented with *hopAH1a (*cloned under the *avrRps4* promoter) | This study. |
| Psa3 V-13 Δ33E + pBBR1MCS-5:*hopAI1b* | Plasmid-complemented with *hopAI1b (*cloned under the *avrRps4* promoter) | This study. |
| Psa3 V-13 Δ33E + pBBR1MCS-5:*hopAM1a* | Plasmid-complemented with *hopAM1a (*cloned under the *avrRps4* promoter) | This study. |
| Psa3 V-13 Δ33E + pBBR1MCS-5:*hopD2a* | Plasmid-complemented with *hopD2a (*cloned under the *avrRps4* promoter) | This study. |
| Psa3 V-13 Δ33E + pBBR1MCS-5:*hopAS1b* | Plasmid-complemented with *hopAS1b (*cloned under the *avrRps4* promoter) | This study. |
| Psa3 V-13 Δ33E + pBBR1MCS-5:*hopAU1a* | Plasmid-complemented with *hopAU1a (*cloned under the *avrRps4* promoter) | This study. |
| Psa3 V-13 Δ33E + pBBR1MCS-5:*hopAT1e* | Plasmid-complemented with *hopAT1e (*cloned under the *avrRps4* promoter) | This study. |
| Psa3 V-13 Δ33E + pBBR1MCS-5:*hopAW1a* | Plasmid-complemented with *hopAW1a (*cloned under the *avrRps4* promoter) | This study. |
| Psa3 V-13 Δ33E + pBBR1MCS-5:*hopAB1ak* | Plasmid-complemented with *hopAB1ak (*cloned under the *avrRps4* promoter) | This study. |
| Psa3 V-13 Δ33E + pBBR1MCS-5:*hopAZ1a* | Plasmid-complemented with *hopAZ1a (*cloned under the *avrRps4* promoter) | This study. |
| Psa3 V-13 Δ33E + pBBR1MCS-5:*hopD1a* | Plasmid-complemented with *hopD1a (*cloned under the *avrRps4* promoter) | This study. |
| Psa3 V-13 Δ33E + pBBR1MCS-5:*hopF1c* | Plasmid-complemented with *hopF1c (*cloned under the *avrRps4* promoter) | This study. |
| Psa3 V-13 Δ33E + pBBR1MCS-5:*hopH1a* | Plasmid-complemented with *hopH1a (*cloned under the *avrRps4* promoter) | This study. |
| Psa3 V-13 Δ33E + pBBR1MCS-5:*hopI1c* | Plasmid-complemented with *hopI1c (*cloned under the *avrRps4* promoter) | This study. |
| Psa3 V-13 Δ33E + pBBR1MCS-5:*hopQ1a* | Plasmid-complemented with *hopQ1a (*cloned under the *avrRps4* promoter) | This study. |
| Psa3 V-13 Δ33E + pBBR1MCS-5:*hopR1b* | Plasmid-complemented with *hopR1b (*cloned under the *avrRps4* promoter) | This study. |
| Psa3 V-13 Δ33E + pBBR1MCS-5:*hopY1b* | Plasmid-complemented with *hopY1b (*cloned under the *avrRps4* promoter) | This study. |
| Psa3 V-13 Δ33E + pBBR1MCS-5:*hopBP1a* | Plasmid-complemented with *hopBP1a (*cloned under the *avrRps4* promoter) | This study. |
| Psa3 V-13 Δ33E + pBBR1MCS-5:*hopZ5a* | Plasmid-complemented with *hopZ5a (*cloned under the *avrRps4* promoter) | This study. |
| Psa3 V-13 Δ33E + pBBR1MCS-5:ShcF:*hopF1e* | Plasmid-complemented with ShcF:*hopF1e (*cloned under the *avrRps4* promoter) | This study. |
| Psa3 V-13 Δ33E + pBBR1MCS-5:ShcF:*hopF4a* | Plasmid-complemented with ShcF:*hopF4a (*cloned under the *avrRps4* promoter) | This study. |
| Psa3 V-13 Δ33E + pBBR1MCS-5:ShcN:*hopN1a* | Plasmid-complemented with ShcN:*hopN1a (*cloned under the *avrRps4* promoter) | This study. |
| Psa3 V-13 Δ33E + pBBR1MCS-5:ShcS2:hopS2b | Plasmid-complemented with ShcS2:hopS2b (cloned under the *avrRps4* promoter) | This study. |
| Psa3 V-13 Δ33E + pBBR1MCS5:*hopBN1a* | Psa3 V-13 Δ33E + pBBR1MCS5:*hopBN1a (*cloned under the *avrRps4* promoter) | This study. |
| Psa3 V-13 Δ33E + pBBR1MCS5:shcM:*hopM1f* | Psa3 V-13 Δ33E + pBBR1MCS5:shcM:*hopM1f (*cloned under the *avrRps4* promoter) | This study. |

**Supplementary Table 2. Psa3 knockout primers used in this study.**

| **Primer** | **Target** | **Primer sequence (5′ – 3′)** |
| --- | --- | --- |
| LH-006 F | *∆avrPto1b* | AGTGTTGGTCAATTAATGTCTCGA |
| LH-006 R | *∆avrPto1b* | ATTGAGACTGCGGCTCTAGA |
| LH-008 F | *∆avrRpm1a* | GCCACAGATCAGTTCCATCTAGA |
| LH-008 R | *∆avrRpm1a* | GGGTAGGCAGCAAAGGACTG |
| LH-024 F | *∆hopAU1a* | TTCGAAGCTGTCTTTGGAACC |
| LH-024 R | *∆hopAU1a* | ACCAATGTGAAACGGGAATCTAGA |
| LH-026 F | *∆hopAZ1a* | TTTCAACTGATTGCCACCAAGAC |
| LH-026 R | *∆hopAZ1a* | GCGGTTGATCATGCGTCTAGA |
| LH-028 F | *∆hopBN1a* | AAAGCGATGGTTTCAGGTGAG |
| LH-028 R | *∆hopBN1a* | GCTCATTGGCACGCTCTAGA |
| LH-030 F | *∆hopF1c* | TCCACAGCATGACCAACAGT |
| LH-030 R | *∆hopF1c* | TGCGGTCGATCAAAATCTCTAGA |
| LH-044 F | *∆hopZ5a/∆hopH1a* | ATAATTTCACATCATGGACGGAACC |
| LH-044 R | *∆hopZ5a/∆hopH1a* | ACTTACAGTAGGTTGCCTCTAGA |
| LH-051-F | *∆hrcC* | TGATGCAAACCACTATTCGTAACAC |
| LH-051-R | *∆hrcC* | TGGCCATTTTAGTTTAAGTTTAGTGAGC |
| LH-061 F | *∆avrRpm1a* | CGCAGTCAATCGCTTCCAG |
| LH-062 R | *∆CEL* | ACGCGTTGCTGAATCCTTTC |
| LH-063 F | *∆fEEL* | GCGCAACTGAACTAGAAAGGG |
| LH-063 R | *∆fEEL* | ATACCGCAGTCCTTCACCTT |
| LH-064 F | *∆hopW1c* | ATCGAGCGCCACTTAGTCC |
| LH-064 R | *∆hopW1c* | AGTTGAAGGGTGGGTGTGAA |
| LH-066 F | *∆hopAS1b* | ACCACCACCTGTATTGATCTGT |
| LH-066 R | *∆hopAS1b* | CAACTGGTGAAGCTGGTGAA |
| LH-071 F | *∆hopI1c* | GATCTCGTTGCTTGCCACAA |
| LH-071 R | *∆hopI1c* | AGTCTGGCTGCCGATCAATA |
| LH-073 F | *∆hopR1b* | TGGCGAGAGTGTCATTAACG |
| LH-073 R | *∆hopR1b* | TGCTGGTGAACTTTGGCA |
| LH-074 F | *∆hopS2b* | CAGCGACAACAACAGCCAAT |
| LH-074 R | *∆hopS2b* | AAGGGCGACTACTTCACCAA |
| LH-076 F | *∆hopBP1a* | AACAGACGTCAGGGTACTC |
| LH-076 R | *∆hopBP1a* | CGATGGGTGCTAGAAACTCTG |
| LH-078 F | *∆sEEL* | CTGGGTAAAACTGGCGCTTT |
| LH-078 R | *∆sEEL* | ATGCAGCGGAGTCAAACAGA |
| LH-079 F | *∆xEEL* | GCCACACAGCAGGTTCATC |
| LH-079 R | *∆xEEL* | ATACCGCAGTCCTTCACCTT |
| LH-080 F | *∆IS* | ACTACTTCACCCAGGACCTG |
| LH-080 R | *∆IS* | CGTTTGCACCAACATCTTCG |
| LH-093 F | *∆hopAM1a* | TCAATGGTGAGGCAAGTGTC |
| LH-093 R | *∆hopAM1a* | ATTTGTAGCAGCAGTTTGTTCTAGA |
| LH-094 F | *∆hopY1b* | AGCAGCGATAAAGGGAGTCT |
| LH-094 R | *∆hopY1b* | CTTGAAGGCCGATGCTACAA |
| LH-098 F | *∆hopQ1a* | GCGTTGCTTGCCACACA |
| LH-098 R | *∆hopQ1a* | CGTATCAAGACCTGGCTGTG |
| LH-099 F | *∆hopD1a* | CGAGGGTAGCACGGTGAAA |
| LH-099 R | *∆hopD1a* | CCATGTCAGTGTGTGCTTCC |
| Psa F1 | ITS | TTTTGCTTTGCACACCCGATTTT |
| Psa R2 | ITS | CACGCACCCTTCAATCAGGATG |
